# Molecular Dynamics Insights into *Cassia tora*-Derived Phytochemicals as Dual Insecticidal and Antifungal Agents Against Tomato *Tuta absoluta* and *Alternaria solani*

**DOI:** 10.3390/ijms27031410

**Published:** 2026-01-30

**Authors:** Tijjani Mustapha, Nathaniel Luka Kwarau, Rajesh B. Patil, Huatao Tang, Mai-Abba Ishiyaku Abdullahi, Sheng-Yen Wu, Youming Hou

**Affiliations:** 1 State Key Laboratory of Ecological Pest Control for Fujian and Taiwan Crops, Key Laboratory of Biopesticides and Chemical Biology, MOE, College of Plant Protection, Fujian Agriculture and Forestry University, Fuzhou 350002, China; tijjani.m@fud.edu.ng (T.M.);; 2 Department of Plant Biology, Federal University Dutse, Dutse P.O. Box 7156, Jigawa State, Nigeria; 3 Department of Pharmaceutical Chemistry, Sinhgad Technical Education Society’s, Sinhgad College of Pharmacy, Vadgaon (Bk), Pune 411041, Maharashtra, India

**Keywords:** *Cassia tora*, squalene, docosahexaenoic acid methyl ester, *T. absoluta*, *A. solani*, molecular dynamics, MM-GBSA, ProLIF, biopesticide, plant-derived pesticide

## Abstract

The pressing need for sustainable, plant-based alternatives is highlighted by the growing resistance of agricultural pests to synthetic pesticides. This study examined the pesticidal potential of phytocompounds from *C. tora* discovered by GC–MS analysis against important tomato insect (*T. absoluta*) and fungal pathogen (*A. solani*). The binding stability and interaction dynamics of specific metabolites with fungal virulence (polygalacturonase, MAP kinase HOG1, and effector AsCEP50) and insect neuromuscular (ryanodine receptor and sodium channel protein) targets were assessed using molecular docking and 100 ns molecular dynamics simulations. Among the screened compounds, squalene and 4,7,10,13,16,19-docosahexaenoic acid, methyl ester (DHAME) exhibited the strongest binding affinities and conformational stability, with MM-GBSA binding free energies of −38.09 kcal·mol^−1^ and −52.81 kcal·mol^−1^ for squalene complexes in *T. absoluta* and *A. solani*, respectively. Persistent hydrophobic and mixed hydrophobic–polar contacts that stabilised active-site residues and limited protein flexibility were found by ProLIF analysis. These lively and dynamic profiles imply that DHAME and squalene may interfere with calcium signalling and stress-response pathways, which are essential for the survival and pathogenicity of pests. Hydrophobic interactions were further confirmed as the primary stabilising force by the preponderance of van der Waals and nonpolar solvation energies. The findings show that *C. tora* metabolites, especially squalene and DHAME, are promising environmentally friendly biopesticide candidates that have both insecticidal and antifungal properties. Their development as sustainable substitutes in integrated pest management systems are supported by their stability, binding efficacy and predicted biosafety.

## 1. Introduction

Tomatoes (*Solanum lycopersicum*) hold significant global importance due to their widespread consumption, nutritional value, economic impact, and culinary versatility, serving as a staple ingredient in numerous cuisines worldwide and playing a vital role in both human nutrition and the economy globally [1,2,3]. China, along with other Asian countries such as Turkey and India, stands as the leading producer of tomatoes [4], whereas among the West African countries, Nigeria appeared highest, with the central regions showing the highest output of 100–200 megatons [5]. These high-production areas, particularly in Nigeria, likely benefit from favourable agricultural conditions, including suitable soil, climate, and access to necessary resources. Despite this significance, the crop faces numerous challenges, including viral diseases, pest infestations, and environmental stresses, which remain the major constraints to global tomato cultivation [6,7]. The perishable nature of this vegetable also adds to these problems. Pests like *Tuta absoluta* (tomato leaf miner) and *Alternaria solani* (tomato early blight agent), common biotic challenges in tomato farming, both lead to substantial yield losses, especially in developing countries, necessitating innovative, effective and sustainable pest management strategies [8].

The major and primary pest management practice in tomato cultivation is the use of synthetic pesticides (insecticides and fungicides), which faces significant challenges that span ecological, health, economic, and regulatory domains. Synthetic pesticides, particularly insecticides targeting pests like *T. absoluta*, are increasingly ineffective due to rapid resistance development. For example, *T. absoluta* populations in Kenya and Uganda have shown resistance to pyrethroids and organophosphates, necessitating higher application frequencies and doses to achieve minimal control [8]. While on the other hand, fungicide resistance in *A. solani* populations is prevalent and evolving rapidly, particularly resistance to succinate dehydrogenase inhibitor (SDHI) fungicides and quinone outside inhibitor (QoI) fungicides. Multiple distinct SDHI resistance-conferring mutations (notably SdhB-H278Y, SdhC-H134R, and D123E) have independently emerged and spread widely across developed nations such as Europe and the United States, with resistance frequencies reaching up to 80% in some populations, linking to significant reductions in fungicide efficacy both in vitro and in field conditions [9,10,11,12]. In addition, synthetic pesticides contribute to soil degradation, water contamination, and non-target species mortality [13], as well as farmers’ and consumers’ health risks as a result of exposure to pesticides such as organophosphates and carbamates. Economic and regulatory gaps also raised another concern, where smallholder farmers in low-income countries often lack access to safer alternatives or integrated pest management (IPM) training. In Cambodia, for instance, farmers spent 30–40% of tomato production costs on synthetic pesticides, yet yields remained low due to pest resurgence [14]. Regulatory frameworks in regions like sub-Saharan Africa are weak; even in regulated markets, inconsistent enforcement and counterfeit products undermine safety [15]. This collectively necessitates the transitioning from the use of synthetic chemical pesticides to sustainable practices that require multidisciplinary collaboration and innovation in pest-specific solutions.

In the efforts of mitigating the effects caused by synthetic pesticides, plant-derived compounds present a transformative solution to the dual challenges of insect and fungal management in tomato cultivation by offering eco-friendly alternatives to synthetic pesticides while maintaining or improving efficacy. Thus, the integration of botanical pesticides into integrated pest management (IPM) systems could address key issues such as pesticide resistance, environmental contamination, and non-target toxicity. Whereas synthetic pesticides have led to pest resistance (e.g., *T. absoluta* developing resistance to 52 active ingredients globally), plant-derived compounds such as essential oils (e.g., citrus peel nanoemulsions, alkaloids, and flavonoids) provide biodegradable alternatives with lower ecological footprints [16,17]. For instance, neem (*Azadirachta indica*) extracts reduced *T. absoluta* larval populations by 60–70% in field trials [18,19], while garlic (*Allium sativum*) extracts disrupted pest oviposition and feeding behaviour [19]. Likewise, treatment of tomato plants with the organic extract significantly reduced the severity of early blight, with recorded disease control efficacy exceeding 90% in two independent greenhouse experiments [20]. This suggests the potential use of botanicals as potential substitutes for the use of toxic pesticides in the management of tomato pests. However, most of the pesticides have a single target mechanism of action, with a higher likelihood of insecticides targeting only insect pests and fungicides targeting only fungal pathogens. This also requires an additional budget by the farmers, which in turn increases the cultivation cost and adds more stress to the environment as a result of the multiple use of these pesticides. This further raised the demand for exploring sustainable broad-spectrum botanical pesticides with multi-target effects against a wide range of pests that are of different biological groups.

As plant-derived secondary metabolites are rich sources of bioactive molecules, molecular docking and molecular dynamics (MD) simulations have revolutionised the discovery of plant-derived agrochemicals by enabling precise predictions of ligand–target interactions, accelerating virtual screening, and optimising lead compounds. Through these techniques, the mechanism of action would also be predicted efficiently in a less time-consuming manner and with minimal resources compared to the traditional approaches. For instance, docking of tomato leaf extracts against *Helicoverpa armigera* detoxification enzymes (e.g., cytochrome P450s) highlighted sesquiterpene lactones as potent inhibitors, validated by MD stability analysis [21]. Subsequently, virtual screening of phenolic acids from tomato rhizosphere microbes against *Fusarium oxysporum* proteases identified rosmarinic acid as a lead compound, with MD confirming its prolonged binding to catalytic sites [22]. Further, simulations of carbendazim (a synthetic fungicide) binding to β-tubulin revealed competitive inhibition mechanisms, guiding the search for plant-derived analogues with similar binding profiles [23]. Thus, following the earlier reported escalating resistance of *T. absoluta* and *A. solani* to synthetic pesticides, coupled with the environmental and health risks, this study aims to screen the phytochemical composition of *C. tora* leaf extract using GC-MS and conduct in silico molecular docking and dynamics simulations to evaluate the binding affinity of the identified compounds against key protein targets in the mentioned detrimental pests affecting tomato. We hypothesised that *C. tora* phytochemicals will exhibit strong inhibitory interactions with the selected receptors, disrupting pest survival mechanisms while maintaining low toxicity to non-target organisms.

## 2. Results

### 2.1. Gas Chromatography-Mass Spectrometry

GC-MS analysis of the methanolic extract of *C. tora* leaves identified 38 bioactive compounds, with fatty-acid derivatives dominating the profile as presented in Table 1. From the result, the most abundant constituents included cis-5,8,11,14,17-eicosapentaenoic acid (13.59%, RT: 30.25 min), 4,7,10,13,16,19-docosahexaenoic acid methyl ester (15.27%, RT: 33.32 min), and squalene (1.31%, RT: 37.85 min). Notable minor compounds comprised butylated hydroxytoluene (0.20%, RT: 13.64 min), dibutyl phthalate (0.68%, RT: 23.59 min), and diethyl phthalate (1.71%, RT: 33.87 min).

### 2.2. Binding Energy Analysis of C. tora Phyto-Compounds Against the Target Proteins of T. absoluta and A. solani

#### 2.2.1. Docking Against *T. absoluta* Targets

**Figure 1:**
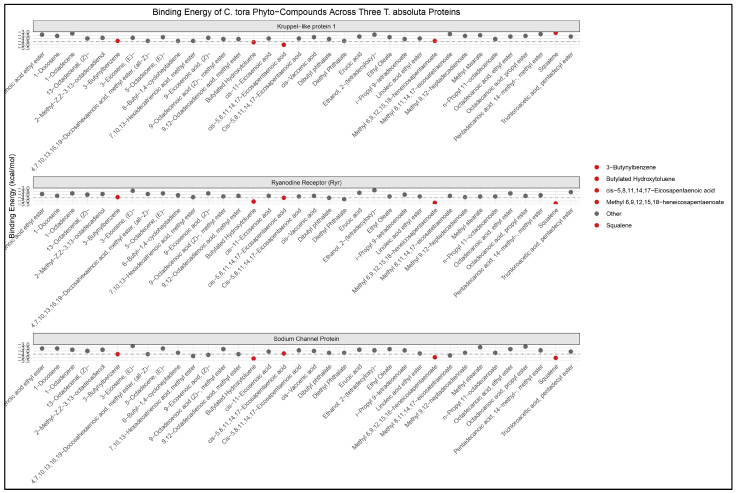
Binding Affinities (ΔG, kcal/mol) of *C. tora* Bioactive Compounds Against *T. absoluta* Target Proteins (Kruppel Protein, Ryanodine Receptor, and Voltage-Gated Na^+^ Channel). ΔG values in red indicate phytochemicals with the highest binding energy to each protein, respectively.

When 35 phytocompounds from *Cassia tora* were docked against three *T. absoluta* proteins—the voltage-gated sodium channel, ryanodine receptor, and Krupple protein—binding energies ranging from −5.4 to −3.1 kcal mol^−1^ were obtained (Figure 1, Table S1).

Predicted affinities for the Kruppel protein varied from −5.0 kcal mol^−1^ to −3.1 kcal mol^−1^. The strongest predicted binding to Kruppel was exhibited by cis-5,8,11,14,17-eicosapentaenoic acid (ΔG = −5.0 kcal mol^−1^), butylated hydroxytoluene (BHT, ΔG = −4.6 kcal mol^−1^), and 3-butynylbenzene (ΔG = −4.4 kcal mol^−1^). In general, affinities were higher against the ryanodine receptor. The lowest (most favourable) ΔG was 5.4 kcal mol^−1^ for squalene, 5.3 kcal mol^−1^ for methyl 6,9,12,15,18-heneicosapentaenoate, and 5.1 kcal mol^−1^ for BHT. Several long-chain methyl esters and phthalate esters also scored below −4.5 kcal mol^−1^ for this receptor.

Out of the three *T. absoluta* targets, the voltage-gated Na^+^ channel displayed the best single-ligand predictions (Figure 1). Again, the BHT ranked highest (ΔG = −5.2 kcal mol^−1^). Closely thereafter were methyl 6,9,12,15,18-heneicosapentaenoate (ΔG = −5.0 kcal mol^−1^) and squalene (ΔG = −5.1 kcal mol^−1^). Intermediate affinities (−4.0 to −4.8 kcal mol^−1^) were produced by a subset of fatty-acid methyl and ethyl esters and phthalate esters. However, these findings show that a select few lipophilic substances, most notably BHT, squalene, and long-chain polyunsaturated methyl esters, continuously produced the best docking scores against *T. absoluta* receptors (Figure 1, Table S1).

#### 2.2.2. Docking Against *A. solani* Targets

**Figure 2:**
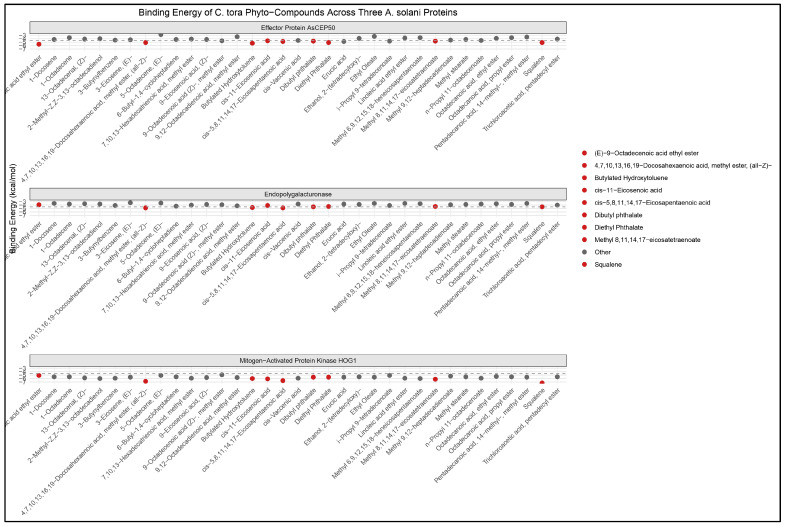
Binding Affinities (ΔG, kcal/mol) of *C. tora* Bioactive Compounds Against *A. solani* Target Proteins (Effector Protein, Endopolygalacturonase, and Mitogen-Activated Protein). ΔG values in red indicate phytochemicals with the highest binding energy to each protein, respectively.

Docking against three *A. solani* proteins (effector protein, endopolygalacturonase, and a mitogen-activated protein kinase, MAP) returned a wider span of binding energies, from −7.3 to −2.8 kcal mol^−1^ (Figure 2, Table S2).

The most favourable predicted binder for the effector protein was (E)-9-octadecenoic acid ethyl ester (ΔG = −5.6 kcal mol^−1^). Significant affinity was also demonstrated by butylated hydroxytoluene (ΔG = −5.3 kcal mol^−1^), 4,7,10,13,16,19-docosahexaenoic acid methyl ester (ΔG = −5.1 kcal mol^−1^), diethyl phthalate (ΔG = −5.1 kcal mol^−1^), and squalene (ΔG = −5.1 kcal mol^−1^). Overall, endopolygalacturonase formed modest affinities; the strongest ones were cis-5,8,11,14,17-eicosapentaenoic acid and 4,7,10,13,16,19-docosahexaenoic acid methyl ester (both having ΔG = −4.8 kcal mol^−1^). For this enzyme, a number of phthalate esters, BHT, and squalene had scores ranging from −4.7 to −4.4 kcal mol^−1^.

Among the targets of *A. solani*, the MAP kinase produced the strongest affinities. Out of all the targets in this investigation, squalene showed the strongest predicted binding (ΔG = −7.3 kcal mol^−1^) (Figure 2). Other high-affinity ligands for MAP included the docosahexaenoic acid methyl ester (ΔG = −6.8 kcal mol^−1^), cis-5,8,11,14,17-eicosapentaenoic acid (ΔG = −6.6 kcal mol^−1^), methyl 8,11,14,17-eicosatetraenoate (ΔG = −6.2 kcal mol^−1^) and cis-11-eicosenoic acid (ΔG = −6.1 kcal mol^−1^). According to these findings, the highest-scoring ligands for *A. solani* MAP are squalene and a number of long-chain polyunsaturated esters. A partially overlapping group of lipophilic compounds demonstrated moderate to strong predicted affinities for the effector protein and endopolygalacturonase (refer to Table S2).

By contrast, the most favourable docking scores were obtained by lipophilic, long-chain fatty acid esters and specific phthalate or antioxidant compounds. Squalene had the most favourable ΔG (−7.3 kcal mol^−1^ for *A. solani* MAP), while butylated hydroxytoluene and squalene consistently scored highly against both insect and fungal proteins. These best candidate compounds were selected for an in-depth molecular interaction’s visualisation and molecular dynamic simulations to ascertain their dynamics over time. For *T. absoluta* targets, the overall binding-energy ranges were −5.4 to −3.1 kcal mol^−1^, while for *A. solani* targets, they were −7.3 to −2.8 kcal mol^−1^ (complete data in Tables S1 and S2).

### 2.3. Residue-Level Interaction Profiles of Top C. tora Phyto-Compounds with T. absoluta and A. solani Targeted Proteins

#### 2.3.1. *T. absoluta*

The interaction profile for the three *T. absoluta* targets shows a consistent pattern of binding for the phytocompounds selected for further study. The compounds illustrated are butylated hydroxytoluene (BHT), 4,7,10,13,16,19-docosahexaenoic acid methyl ester (DHAME), and squalene. These complexes were chosen because each ligand produced uniformly favourable docking scores across the *T. absoluta* receptors (Figure 1, Table S1).

**Figure 3:**
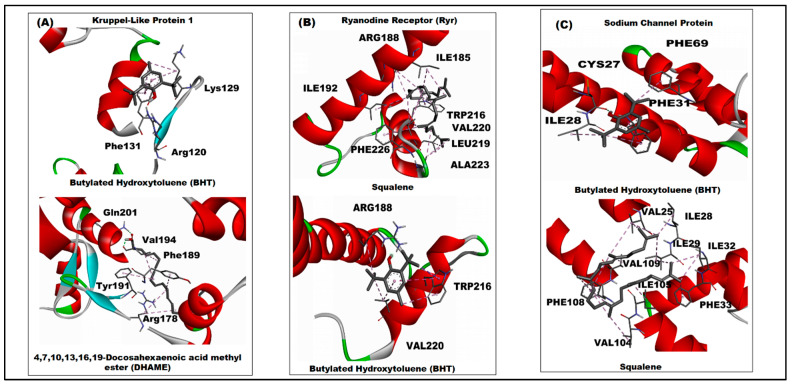
3D interaction of *C. tora* phytocompounds with uniform favourable docking scores across the *T. absoluta* receptors. (**A**) Kruppel-like Protein 1; (**B**) Ryanodine Receptor (Ryr); and (**C**) Sodium Channel Protein.

The Kruppel pocket accommodates both butylated hydroxytoluene (BHT) and DHAME, but each ligand exploits the site differently (Figure 3A). BHT is positioned against a cluster of aromatic and hydrophobic side chains. The 2D map (Figure S1) indicates multiple aromatic contacts consistent with pi–alkyl and pi–pi-type interactions involving phenylalanine residue (PHE131) and extensive alkyl/van der Waals contacts with LYS129, ILE132 and THR130 side chains. DHAME inserts its long hydrocarbon tail into the same hydrophobic groove, producing broad van der Waals and alkyl contacts, while the ester oxygen of DHAME forms one or two polar contacts with nearby backbone or side-chain donors. Interestingly, the result indicates a mixed binding mode where shape complementarity and hydrophobic packing dominate, and where discrete polar contacts at the ligand termini likely help anchor orientation.

However, squalene occupies an extended lipophilic cleft in the ryanodine receptor (Figure 3B). The 2D diagram (Figure S2A) shows a continuous run of alkyl/van der Waals interactions with nonpolar residues, notably multiple isoleucine (ILE185, ILE192), valine (VAL220, VAL228) and leucine (LEU219) contacts that create an elongated hydrophobic interface. BHT binds nearby and displays aromatic stacking and alkyl contacts; the BHT ring system engages TRP216 and other hydrophobic residues to stabilise its pose. Neither ligand forms dense hydrogen-bond networks; instead, the interactions are dominated by nonpolar contacts and close packing (Figure S2A,B).

For the sodium channel (Figure 3C), the results show BHT and squalene lodged within a predominantly hydrophobic pocket formed by branched-chain aliphatic residues. The interactions (Figure S3A) indicated van der Waals and alkyl contacts with several residues (PHE69, ILE28, PHE65, PHE24, ILE30) along the channel surface and indicated limited polar contacts at ligand termini. A conventional hydrogen bond was observed with CYS27. In the BHT complexes the aromatic core is orientated to permit close contacts with aromatic side chains, consistent with pi–alkyl stabilisation (Figure 3C and Figure S3B). The overall interaction pattern mimics the other *T. absoluta* targets: dominant hydrophobic packing with occasional polar interaction (Figures S1 and S2).

Across the three insect proteins, the residue-level interactions with *C. tora* compounds are dominated by hydrophobic and aromatic side chains. Phenylalanine, leucine and valine residues recur as principal contact partners. Polar residues supply occasional hydrogen bonds or polar contacts, typically at ligand termini (the ester oxygen of DHAME is the most consistent polar anchor). These residue-level profiles support the conclusion that lipophilic packing drives the favourable docking scores reported in Figure 1 and Table S1.

#### 2.3.2. *A. solani*

**Figure 4:**
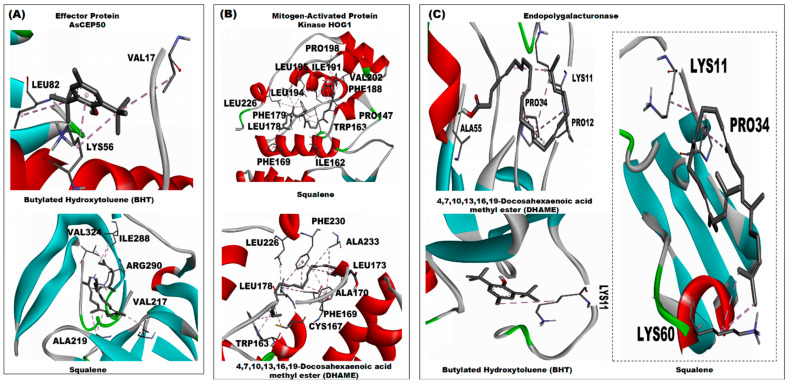
3D interaction of *C. tora* phytocompounds with uniform favourable docking scores across the *A. solani* receptors. (**A**) Effector Protein AsCEP50; (**B**) Mitogen-Activated Protein Kinase HOG1; and (**C**) Endopolygalacturonase.

The effector protein surface binds BHT and squalene mainly through nonpolar residues (Figure 4A). The 2D interaction (Figure S4A) indicates alkyl interactions with LEU82, LYS56 and VAL17 and van der Waals contacts with GLY19, ALA52, ARG53, ALA57, VAL60 and GLY99 side chain interactions with the BHT phytocompound. Polar or charged residues are not prominent in the annotated contact list for these complexes, suggesting that dispersive interactions and surface complementarity are the principal stabilising forces (Figure S4A,B). The endopolygalacturonase reveals a mixed interaction profile (Figure 4C and Figure S5A–C). DHAME combines deep hydrophobic insertion via its hydrocarbon tail with specific polar contacts through the ester oxygen. The 2D map (Figure S5A) indicates that the ester oxygen approaches hydrogen-bond donors or polar side chains (serine, threonine or backbone amides) in the catalytic vicinity, while the tail is cradled by lysine and proline residues. BHT and squalene (Figure S5B,C) again show dominant hydrophobic and aromatic contacts. These residue-level features imply that DHAME may interact both by occupying a hydrophobic patch and by forming discrete polar contacts that could influence substrate access or local conformation.

The MAP kinase binding cleft presents the most extensive annotated contact network among the fungal targets (Figure 4B). Squalene is shown making an extended series of van der Waals and alkyl contacts with a contiguous stretch of hydrophobic residues, producing high shape complementarity (Figure S6A). DHAME presents a hybrid pattern in the MAP interaction; the hydrocarbon tail binds hydrophobic side chains, while the ester oxygen participates in one or more polar contacts with side-chain or backbone donors. Aromatic residues also appear among the annotated contacts, providing pi-type stabilisation for BHT where present (Figure 4B and Figure S6B). The observed residue interaction for MAP corresponds with the comparatively favourable docking energies recorded for this target in Figure 2 above. In the fungal targets the residue-level interactions show the same dominant motif, where hydrophobic and aromatic residues provide an extensive contact surface while ester-containing ligands add targeted polar interactions. MAP kinase displays the densest network of contacts, consistent with its most favourable docking scores.

### 2.4. Molecular Dynamics Simulations and MM-GBSA Calculations

To gain atomic-level insights into the dynamic stability, conformational flexibility, and binding energies of *C. tora*–derived phytocompounds against key target proteins of *T. absoluta* (insect) and *A. solani* (fungus), trajectories from 100 ns were analysed for Root Mean Square Deviation (RMSD), Root Mean Square Fluctuation (RMSF), Radius of Gyration (Rg), hydrogen-bond dynamics, buried solvent-accessible surface area (*B-SASA*), and Molecular Mechanics/Generalised Born Surface Area (MM/GBSA) energy components to evaluate conformational stability and binding efficiency.

#### 2.4.1. Dynamic Behaviour of *T. absoluta* and *A. solani* Protein–Ligand Complexes

##### Conformational Stability from RMSD

**Figure 5:**
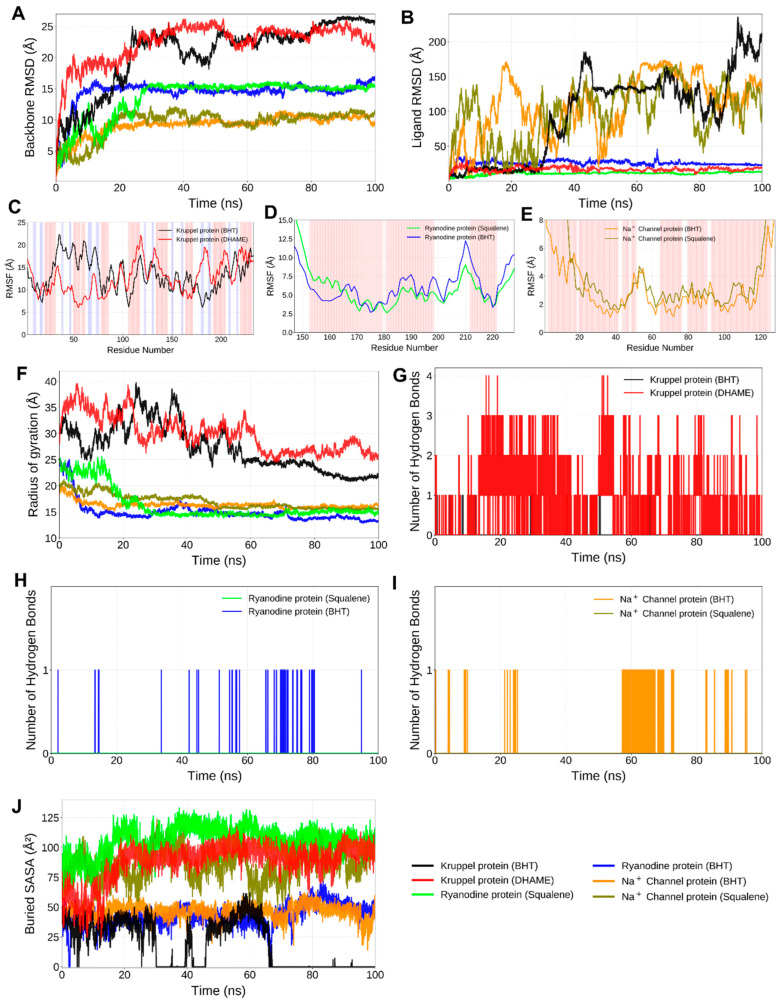
RMSD, RMSF, radius of gyration, H-bond analysis and buried *SASA* calculations for *T. absoluta* (Kruppel-like protein 1, ryanodine receptor, and sodium channel protein). (**A**) RMSD in protein backbone atoms, (**B**) RMSD in ligand atoms relative to protein backbone atoms, (**C**) RMSF in Kruppel-like protein 1 side chains, (**D**) RMSF in Ryanodine receptor side chains, (**E**) RMSF in Sodium Channel Protein, (**F**) Radius of gyration, (**G**) H-bonds formed between Kruppel-like protein 1 and ligands, (**H**) H-bonds formed between Ryanodine receptor and ligands, (**I**) H-bonds formed between Sodium Channel Protein and ligands, and (**J**) Buried solvent accessible surface area plot. (The abbreviations used for the ligands in the plots are BHT: butylated hydroxytoluene and DHAME: 4,7,10,13,16,19-docosahexaenoic acid, methyl ester).

Indicating stable conformational equilibrium, the Kruppel-like protein 1 complexes with BHT and DHAME stabilised within the first 10 ns, maintaining mean deviations below 0.20 nm (Figure 5A). Likewise, there was no significant drift during the simulation for the protein complexes of the sodium channel and ryanodine receptor, which showed steady RMSD fluctuations between 0.18 and 0.25 nm. The squalene–ryanodine receptor complex showed the lowest RMSD amplitude (~0.17 nm) of any system, indicating a stable structural conformation and minimal disturbance in its secondary components. The ligand RMSD values closely followed the backbone trends, implying firm retention within the active site cavities and absence of dissociative behaviour (Figure 5B). Collectively, these patterns confirm high dynamic stability and strong conformational adaptability of the phytochemical ligands within their target binding pockets.

**Figure 6:**
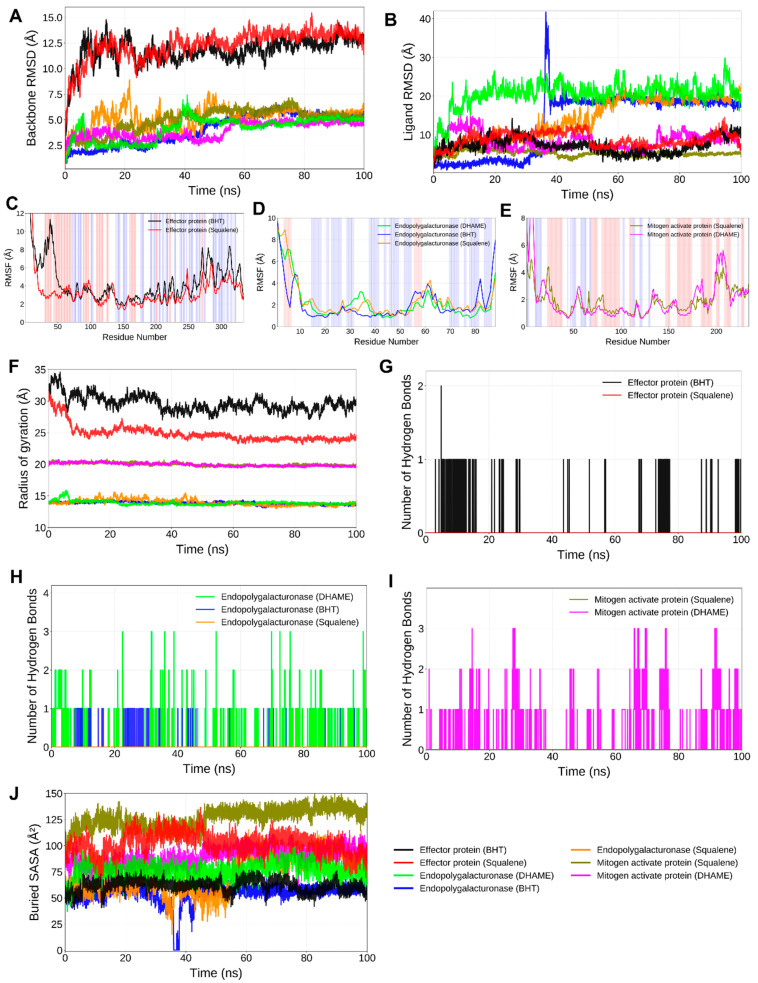
RMSD, RMSF, radius of gyration, H-bond analysis and buried *SASA* calculations for *A. solani* (effector protein AsCEP50, polygalacturonase and mitogen-activated protein kinase HOG1). (**A**) RMSD in protein backbone atoms, (**B**) RMSD in ligand atoms relative to protein backbone atoms, (**C**) RMSF in Effector Protein AsCEP50 side chains, (**D**) RMSF in Polygalacturonase (endopolygalacturonase) side chains, (**E**) RMSF in Mitogen-activated protein kinase HOG1, (**F**) Radius of gyration, (**G**) H-bonds formed between Effector Protein AsCEP50 and ligands, (**H**) H-bonds formed between Polygalacturonase and ligands, (**I**) H-bonds formed between Mitogen-activated protein kinase HOG1 and ligands, and (**J**) Buried solvent accessible surface area plot. (The abbreviations used for the ligands in the plots are BHT: butylated hydroxytoluene and DHAME: 4,7,10,13,16,19-docosahexaenoic acid, methyl ester).

All *A. solani* protein–ligand systems showed consistent stability in RMSD trajectories (Figure 6A,B). With average backbone RMSD values ranging from 0.18 to 0.28 nm, each complex was stabilised for the duration of the 100 ns simulation after reaching equilibrium in 10–15 ns (Figure 6A). The polygalacturonase–DHAME and effector protein AsCEP50–squalene complexes showed the lowest deviation amplitudes, indicating minimal drift from the native backbone structure and strong conformational maintenance. Robust stability was demonstrated by the MAP kinase HOG1–squalene complex, which stabilised quickly with fluctuations below 0.20 nm. Protein trajectories were paralleled by ligand RMSD profiles (Figure 6B), indicating that the binding cavities were persistently occupied and that dissociative motion was absent. These results collectively imply that ligand binding reinforced conformational equilibrium and maintained compact folded states throughout the trajectory.

##### Residue Flexibility from RMSF

Root Mean Square Fluctuation (RMSF) profiles provided residue-level insights into protein flexibility in *T. absoluta* receptors (Figure 5C–E). Fluctuations were typically less than 0.3 nm in all of the simulated systems, indicating restrained side-chain mobility and maintained tertiary architecture. Its capacity to stabilise the local environment was demonstrated by the decreased atomic fluctuations in Kruppel-like protein 1 residues that form the ligand-binding interface, especially in complexes with DHAME (Figure 5C). The residues interacting with squalene remained highly stable, highlighting strong hydrophobic bonding, while the ryanodine receptor showed noticeable flexibility in loop segments far from the binding core (Figure 5D). On the other hand, the BHT–Sodium channel protein displayed a moderate degree of flexibility close to surface loops (Figure 5E), indicating brief breathing motions that did not interfere with global folding. The general RMSF patterns support the rigidity and stabilisation brought about by ligands at catalytically significant regions.

For the *A. solani* complexes, the RMSF analysis identified residue-level fluctuations and binding-site rigidity for each complex (Figure 6C–E). The majority of residues showed limited mobility, fluctuating below 0.30 nm. Squalene binding significantly decreased the flexibility of the residues that form the catalytic pocket and recognition loop in AsCEP50, indicating stronger hydrophobic stabilisation (Figure 6C). In residues that bordered the substrate-binding cleft, polygalacturonase–DHAME complexes showed dampened motion (Figure 6D), which is consistent with efficient ligand anchoring. Both DHAME and squalene inhibited oscillations in the activation-loop region (residues 165–190) of MAP kinase HOG1 (Figure 6E), a region crucial for kinase regulation. These steady RMSF decreases suggest that compounds from *Cassia tora* stiffen important functional domains, which may hinder enzymatic conformational changes necessary for pathogenic activity.

##### Compactness from Radius of Gyration

Radius of gyration (Rg) analysis revealed steady compactness in all *T. absoluta* complexes, with average Rg values ranging from 1.85 to 2.05 nm (Figure 5F). The overall tertiary structures were confirmed to have been well preserved during the 100 ns simulation by the Rg trajectories, which showed few deviations. With the lowest mean Rg, the squalene–ryanodine receptor complex showed the most compact conformation, which is consistent with the secondary structural elements being tightly packed around the hydrophobic ligand. The BHT-Sodium channel protein, on the other hand, showed slightly higher Rg fluctuations, indicating a slight degree of global flexibility that may have been caused by peripheral domain motions. These results, in agreement with RMSD (Figure 5A,B) and RMSF analyses (Figure 5C–E), confirm that ligand association enhances the conformational resilience and compactness of target proteins.

In *A. solani* complexes, throughout the 100 ns simulation, high conformational compactness was confirmed by radius-of-gyration trajectories (Figure 6F). After the initial equilibration period, there were very slight variations in the average Rg values, which varied between 1.80 and 2.05 nm. The HOG1–squalene complex displayed the narrowest fluctuation band and the smallest Rg, indicating secondary-structure arrangements that were densely packed and held in place by hydrophobic ligand interactions. RMSD findings (Figure 6C–E) were supported by the similarly compact profiles of AsCEP50–Squalene and Polygalacturonase–DHAME. The fact that Rg remained stable in all systems confirms that ligand binding prevented domain separation and maintained the integrity of the tertiary structure overall.

##### Hydrogen-Bond and Buried *SASA* Analyses

The crucial function of polar interactions in complex stabilisation was highlighted by hydrogen bond occupancy analysis (Figure 5G–I). Throughout the trajectory, the BHT–Kruppel-like protein 1 complex maintained an average of two stable hydrogen bonds, mostly with the recognition helix’s backbone amide groups (Figure 5G). Although hydrophobic contacts predominated, the squalene–ryanodine receptor complex maintained random hydrogen bonds with polar residues, preserving the ligand’s orientational stability (Figure 5H). In line with the moderate affinity predicted by MM/GBSA energies (see Table 2 below), the BHT–sodium channel protein complex exhibited stable van der Waals interactions along with transient hydrogen bonds (Figure 5I). The squalene–ryanodine receptor showed the largest buried area, indicating deep ligand insertion into a hydrophobic core. Buried *SASA* profiles (Figure 5J) showed significant solvent exclusion upon ligand binding. A favourable enthalpic contribution to complex stabilisation is indicated by the high *B-SASA* values and consistent hydrogen bonding.

Comparatively in *A. solani*, persistent intermolecular contacts were revealed by hydrogen-bond analyses, which significantly enhanced the overall stability of the complex (Figure 6G–I). Two to three continuous hydrogen bonds were maintained by the AsCEP50–BHT complex, primarily involving amide and carbonyl backbone residues close to the effector loop. Squalene, although largely hydrophobic, exhibited intermittent but spatially consistent hydrogen contacts within polar pocket regions of both AsCEP50 and HOG1, supporting orientational stabilisation of the aliphatic chain. The DHAME–polygalacturonase complex reinforced a tight binding geometry by displaying the highest hydrogen-bond occupancy (averaging 3–4 bonds per frame) with residues central to the catalytic groove. Deep ligand burial within hydrophobic cores was indicated by the extensive solvent exclusion for Squalene–HOG1 and DHAME–Polygalacturonase shown in the Buried-SASA plots (Figure 6J). These characteristics collectively validate energetically advantageous enthalpic contributions resulting from hydrophobic encapsulation and persistent hydrogen bonding.

##### Gibbs Free-Energy Landscape (FEL)

**Figure 7:**
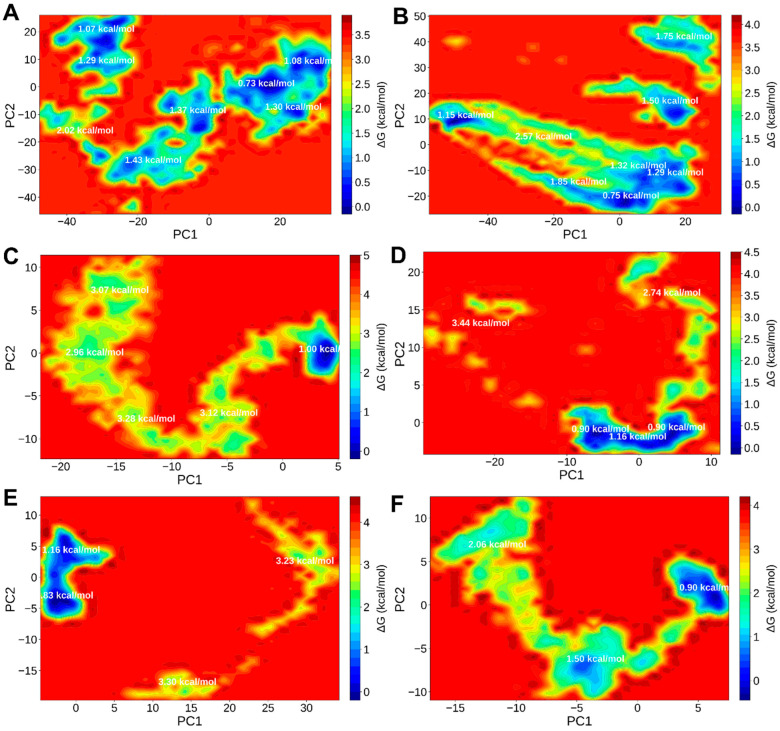
Gibbs Free-Energy Landscapes for complexes of respective ligands with Kruppel-like protein 1, Ryanodine receptor, and Sodium Channel Protein from *T. absoluta*. Complex of Kruppel-like protein 1 with (**A**) butylated hydroxytoluene and (**B**) 4,7,10,13,16,19-docosahexaenoic acid, methyl ester; complex of Ryanodine receptor with (**C**) squalene and (**D**) butylated hydroxytoluene; complex of sodium channel protein with (**E**) butylated hydroxytoluene and (**F**) squalene. (The average energy in kcal/mol is shown in the respective low-energy basins).

Principal component analysis followed by Gibbs free-energy landscape (FEL) mapping revealed the conformational energy topography of the simulated *T. absoluta* complexes (Figure 7A–F). During simulation, the majority of systems showed only one deep basin, indicating few conformational changes. Notably, compact, low-energy funnels with average minima of about −45 kcal/mol were shown by DHAME–Kruppel-like protein 1 and the Squalene–Ryanodine receptor, suggesting thermodynamically stable states. However, a wider landscape with several shallow basins was shown by the BHT–sodium channel protein complex, indicating transient conformer populations and relatively lower stability. Squalene and DHAME are thus highlighted as extremely stable binders by the FEL analyses, which are in agreement with RMSD and MM/GBSA data.

**Figure 8:**
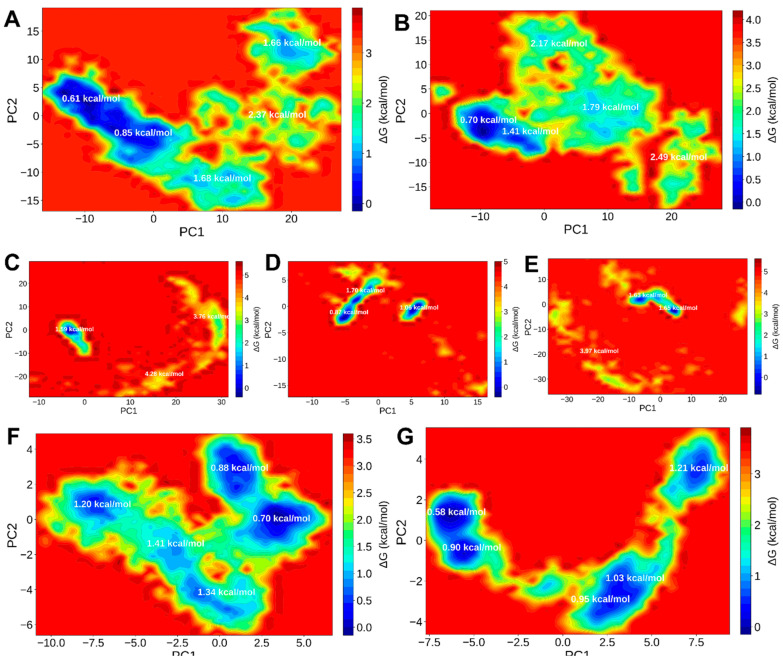
Gibbs Free-Energy Landscapes for complexes of respective ligands with Effector Protein AsCEP50, Polygalacturonase and Mitogen-activated protein kinase HOG1 from *A. solani*. Complex of Effector Protein AsCEP50 with (**A**) Butylated hydroxytoluene, (**B**) Squalene; complexes of polygalacturonase (endopolygalacturonase) with (**C**) 4,7,10,13,16,19-docosahexaenoic acid, methyl ester, (**D**) Butylated hydroxytoluene, (**E**) Squalene; Complex of Mitogen-activated protein kinase HOG1 with (**F**) Squalene, and (**G**) 4,7,10,13,16,19-docosahexaenoic acid, methyl ester.

Further, the principal-component-based FEL mapping delineated the conformational energy minima populated during the trajectories in *A. solani* protein–ligand complexes (Figure 8A–G). Single dominant basins were present in the majority of *A. solani* complexes, indicating convergence towards stable conformational ensembles. Low conformational heterogeneity and high thermodynamic stability were indicated by the deep, narrow energy funnels (ΔG ≈ −45 to −55 kcal/mol) displayed by the DHAME–polygalacturonase and Squalene–HOG1 complexes. The BHT–AsCEP50 complex, on the other hand, showed a wider landscape with several shallow minima, indicating transitory metastable states in line with the moderate stability seen in its MM/GBSA and RMSD profiles. Squalene and DHAME stabilised the lowest-energy conformers of their respective protein targets, according to the FEL patterns overall.

### 2.5. Protein–Ligand Interaction Fingerprinting (ProLIF) Analysis

A protein–ligand interaction fingerprinting (ProLIF) analysis (Figures S7–S14) was carried out on the equilibrated segment of the trajectories (50–100 ns) in order to identify the residue-level contact patterns that underlie the dynamic stability of the simulated complexes. A 3.5 Å distance cutoff and a 120° angle criterion for hydrogen bonds were used to quantify interaction occupancies for the hydrophobic, hydrogen-bond, π–π, and salt-bridge categories. Contacts seen in 30–69% and <30% of frames were categorised as moderate and transient, respectively, whereas contacts seen in ≥70% of frames were categorised as persistent or “core” interactions. Figure 7 and Figure 8 display representative interaction heatmaps and per-residue occupancy plots (Figures S9–S14), while Supplementary Tables S1 and S2 contain complete binding energy data.

#### 2.5.1. ProLIF Profiles of *T. absoluta* Complexes

The *T. absoluta* proteins, namely the sodium channel protein, the ryanodine receptor, and the Krupple-like protein 1 (Figure S7A–F), showed unique contact signatures that reflected the binding-site environment and the chemical makeup of the ligand (Figures S9–S11). With hydrophobic interactions accounting for over 70% of all contacts and dispersed throughout the nonpolar residues that make up the inner channel cavity, the squalene–ryanodine receptor complex showed the most compact and durable contact network. Several aliphatic and aromatic side chains lining the hydrophobic gate region showed persistent contacts and were continuously engaged over the course of the 50–100 ns trajectory. In accordance with its extremely favourable ΔG_binding_ (−38.09 ± 4.99 kcal mol^−1^) and low RMSD noted in Figure 5A, hydrogen-bonding events were uncommon (<20% occupancy) and brief, suggesting that van der Waals and nonpolar forces are the main sources of squalene stabilisation.

The protein complexes of the BHT–Ryanodine receptor (Figures S7D and S10B) and the BHT–Sodium channel (Figures S7E and S11A), on the other hand, displayed inconsistent and transient interaction profiles. Similarly to the wider FEL basins and weaker binding energies (−6.51 kcal mol^−1^ and −1.23 kcal mol^−1^, respectively), their fingerprints showed sporadic hydrogen bonds (30–40% occupancy) along with fluctuating hydrophobic contacts. The idea that BHT interacts more superficially and with less conformational restraint than squalene or DHAME is supported by this transient binding characteristic.

The stabilisation pattern of the DHAME–Kruppel-like protein 1 complex was hybrid (Figures S7B and S9B). One or two moderately stable hydrogen bonds (occupancy ~60%), mostly involving polar residues at the pocket boundary, coexisted with persistent hydrophobic contacts. The amphiphilic structure of DHAME is consistent with this combination of polar anchoring and nonpolar burial, which also explains its stable RMSD/Rg trajectory (Figure 5A,F) and intermediate binding free energy (−2.40 kcal mol^−1^). Importantly, the *T. absoluta* ProLIF results show that BHT depends on weaker, transient hydrogen bonds, while squalene forms the most persistent hydrophobic core interactions. The balanced dual-mode stabilisation of DHAME allows for moderate but constant affinity for a variety of targets.

#### 2.5.2. ProLIF Profiles of *A. solani* Complexes

Contact frequency showed similarly ligand-dependent but more complex interaction architectures for the fungal systems (*A. solani*) (Figure S8A–G). The ATP-binding cleft and activation-loop residues were the sites of extensive hydrophobic occupancy (>75%) in the Squalene–HOG1 MAP kinase complex (Figures S8F and S14A), which formed a continuous nonpolar cluster that lasted for more than 90% of simulation frames. Although they were occasionally observed, hydrogen bonds (less than 25% occupancy) with catalytic-loop residues did not predominate the interaction landscape. The squalene high binding affinity (ΔG_binding = −52.81 ± 5.82 kcal mol^−1^) is primarily determined by hydrophobic contact, as evidenced by its very low RMSD (Figure 6A), narrow Rg distribution (Figure 6F), and deepest FEL (Figure 7) minimum among all simulated systems.

The complementary profile of the DHAME–polygalacturonase complex was defined by a well-balanced combination of polar and hydrophobic interactions (Figure S13A). While nearby hydrophobic residues provided extra stabilisation through van der Waals contacts (occupancy ≥ 60%), two residues inside the catalytic groove created moderately persistent hydrogen bonds (occupancy 55–70%). The resulting fingerprint shows that the polar ester moiety of DHAME mediates directional hydrogen bonds, and its flexible unsaturated chain allows for deep penetration into the enzyme cleft. This structural alignment is consistent with the favourable ΔG_binding_ (−5.92 ± 6.86 kcal mol^−1^) and the observed RMSF (Figure 6C) suppression at catalytic residues.

BHT–AsCEP50 interactions (Figure S12A), on the other hand, were noticeably less stable, showing low overall occupancy and several transient hydrogen bonds that hardly ever lasted past 30% of the trajectory. These results provide an explanation for the effector-protein complex’s moderate binding energy (−18.82 kcal mol^−1^) and higher conformational heterogeneity. Altogether, the *A. solani* ProLIF results support the MM-GBSA and MDS trends; DHAME stabilises catalytic interfaces through a combination of hydrophobic and hydrogen-bonding modes, whereas hydrophobic encapsulation dominates in high-affinity complexes (Squalene–HOG1). The low-occupancy and high-variability patterns of BHT complexes support their relatively weak and ephemeral binding behaviour.

### 2.6. MM/GBSA Binding Free Energy

The energetic contributions promoting ligand association were measured by the MM/GBSA binding free energy analysis (Table 2). Out of all the complexes, the squalene–ryanodine receptor had the best binding free energy (ΔGbinding = −38.09 ± 4.99 kcal/mol), which was primarily regulated by strong van der Waals interactions (ΔVDWAALS = −51.08 kcal/mol). BHT complexes with the Kruppel-like protein 1 and sodium channel protein showed lower overall binding energies (ΔGbinding = +7.88 and −1.23 kcal/mol, respectively), whereas the DHAME–Kruppel-like protein 1 complex showed moderate affinity (ΔGbinding = −2.40 ± 6.07 kcal/mol). Despite partial solvation penalties, the squalene–sodium channel protein complex demonstrated intermediate stability (ΔGbinding = −8.52 ± 8.56 kcal/mol), indicating favourable hydrophobic and van der Waals interactions. Strong binding contributions from the DHAME–Kruppel-like protein 1 and squalene–ryanodine receptor complexes confirm the high binding efficiency of the ligands and dynamic persistence against *T. absoluta* molecular targets.

In the case of *A. solani* complexes, the binding affinities and component energy contributions were also measured using MM/GBSA analysis (Table 3). Van der Waals interactions (ΔVDWAALS = −67.01 kcal/mol) and non-polar solvation energies (ΔESURF = −9.11 kcal/mol) dominated the Squalene–MAP kinase HOG1 complex, which showed the best ΔGbinding (−52.81 ± 5.82 kcal/mol). Closely behind (ΔGbinding = −32.65 ± 4.18 kcal/mol) was the DHAME–HOG1 complex, demonstrating complementary hydrophobic and electrostatic contributions. Although AsCEP50–BHT and AsCEP50–Squalene showed moderate affinities (−18.82 ± 3.73 kcal/mol and −15.59 ± 16.29 kcal/mol), Squalene and DHAME produced binding energies of −11.67 ± 12.14 kcal/mol and −5.92 ± 6.86 kcal/mol, respectively, for polygalacturonase. These findings demonstrate DHAME’s reliable stabilisation across catalytic proteins and emphasise squalene’s superior binding energetics towards the kinase target. The strong van der Waals dominance across systems further confirms the hydrophobic nature of *Cassia tora* phytochemicals as a key stabilising agent.

### 2.7. Toxicity Analysis

The toxicity analysis result in Table 4 classified BHT as Toxicity Class 4 (LD50 = 650 mg/kg), with high probabilities for BBB penetration (0.94) and mitochondrial membrane potential (MMP) disruption (0.96), indicating neurotoxic and oxidative stress risks. Moderate ecotoxicity (0.63) and CYP2C9 inhibition (0.70) were noted, though hepatotoxicity was inactive (0.52). Squalene, on the other hand, was categorized as Toxicity Class 5 (LD50 = 5000 mg/kg), exhibiting strong BBB penetration (0.97) but no discernible mutagenicity or hepatotoxicity. Squalene demonstrated moderate suppression of CYP2C9 (0.67) and activated antioxidant pathways (Nrf2/ARE: 0.60). With similar ecotoxicity probability (BHT: 0.63; squalene: 0.62), both substances were classified as moderately hazardous.

Further, the ECOSAR predictive modelling results, detailed in Table 5, indicated varying environmental fates and aquatic toxicities for the tested compounds. Squalene and DHAME were characterised by exceptionally high predicted log K_ow_ values of 14.122 and 8.905, respectively, suggesting high hydrophobicity and very low water solubility (6.65 × 10^−10^ mg/L and 1.71 × 10^−4^ mg/L, respectively). Consequently, for most aquatic acute endpoints, ECOSAR predicted ‘no effects’ up to the saturation concentration due to insufficient dissolution in the aqueous phase.

Comparatively, BHT had a lower predicted log K_ow_ (5.029) and higher water solubility (5.748 mg/L). While specific standard acute toxicity values (LC50/EC50) were not provided in the ECOSAR output for GHS classification, BHT exhibited the highest predicted dermal absorption dose per event (380.959 mg/cm^2^) and biotransformation half-life of 0.32 days. Squalene and DHAME showed minimal to no biotransformation.

## 3. Discussion

Promising relationships between important metabolites and vital proteins of *A. solani* and *T. absoluta* were found during the investigation of *C. tora* phytochemicals as environmentally friendly substitutes for synthetic pesticides. The study used molecular docking, GC–MS profiling, and molecular dynamics simulations to understand the structural and thermodynamic mechanisms of these interactions.

Among the identified compounds, squalene and 4,7,10,13,16,19-docosahexaenoic acid, methyl ester (DHAME) demonstrated superior binding affinities and dynamic stability toward both insect and fungal targets. The MM-GBSA analysis revealed strong negative ΔGbinding values, such as −38.09 kcal·mol^−1^ for the Squalene–Ryanodine receptor and −52.81 kcal·mol^−1^ for squalene–MAP kinase HOG1, indicating their predicted high inhibitory potential. The dominance of van der Waals and nonpolar solvation contributions demonstrates the critical role of hydrophobic interactions in stabilising these complexes, consistent with previous findings that hydrophobic contact networks often govern ligand–protein affinity in pesticidal interactions [24,25].

The ProLIF analysis confirmed persistent hydrophobic interactions in squalene complexes and a combination of hydrophobic and polar contact networks for DHAME. These structural features likely enhance the conformational rigidity of active-site residues, limiting the functional dynamics of target proteins. Inhibiting the ryanodine receptor and sodium channel protein in *T. absoluta* can disrupt calcium signalling and neuromuscular coordination, potentially causing paralysis and mortality similar to the effects recorded on conventional insecticides [26,27]. In *A. solani*, the stable binding of squalene and DHAME to the MAP kinase HOG1 and polygalacturonase enzymes suggests interference with stress signalling and cell wall degradation pathways, which are crucial for fungal virulence and host invasion [28]. These effects are inferred from stable binding modes, persistent interaction networks, and favourable free energy profiles, as revealed by in silico analyses.

Phytochemicals with high hydrophobic content, such as squalene, are known to penetrate lipid membranes efficiently and alter protein–lipid interactions, thereby exerting broad-spectrum antimicrobial and insecticidal effects [29]. Similarly, the amphiphilic structure of DHAME allows simultaneous hydrophobic insertion and hydrogen bonding, enhancing both binding specificity and persistence within catalytic grooves. These insights suggest that squalene and DHAME are promising candidates for developing natural bioinsecticides and antifungal agents sourced from *C. tora*.

The dynamic stability of these compounds, demonstrated by consistently low RMSD and Rg values during 100 ns simulations, suggests durable ligand retention and minimal conformational drift, further supporting their biostability under physiological conditions. Relatively, butylated hydroxytoluene (BHT) exhibited weaker and transient binding patterns with higher energy fluctuations, indicating limited pesticidal relevance. The differences observed suggest the relationship between structure and activity in *C. tora* metabolites, where molecular flexibility and hydrophobic surface area play significant roles in determining their biological effectiveness.

The ECOSAR results provide crucial indication into the environmental sustainability of the *C. tora*-derived phytochemicals as potential biopesticides. The high lipophilicity (log *K*_*ow*_) observed for squalene and DHAME suggests a low probability of reaching toxic concentrations in the water column in natural aquatic environments. However, these compounds also exhibited high predicted bioconcentration factors (BCF values of 4.31 and 1.55 × 10^3^ L/kg, respectively) and bioaccumulation factors (BAF values of 33.11 and 4.63 × 10^4^ L/kg, respectively), suggesting a potential for accumulation in aquatic organisms and transfer up the food chain if introduced into the environment. This highlights the need for responsible application strategies in the field [30]. On the other hand, BHT presented as the most water-soluble candidate, with a more moderate BCF value of 645.62 L/kg. Its rapid predicted biotransformation half-life (0.32 days) suggests it is less persistent in the environment than the other two compounds. Therefore, these *C. tora* compounds offer favourable ecological profiles compared to many persistent synthetic pesticides, supporting their investigation as ‘green’ alternatives in integrated pest management [31].

Moreover, the biosafety profile of *C. tora* extracts, previously reported as non-toxic and biodegradable [32,33], enhances the feasibility of using squalene- and DHAME-based formulations as sustainable pest-control alternatives. This aligns with the global pursuit of biopesticides that mitigate resistance buildup, reduce chemical residues, and preserve ecological balance [34,35]. The present findings thus not only validate *C. tora* as a valuable reservoir of pesticidal phytochemicals but also demonstrate the utility of molecular simulations in rational biopesticide discovery.

Future studies should combine laboratory tests for enzyme inhibition and real-world effectiveness to confirm the predicted mechanisms and investigate combining squalene and DHAME with microbial or botanical carriers for enhanced effects. Additionally, structure-guided optimisation using quantitative structure–activity relationship (QSAR) and machine learning models may enhance their potency and selectivity against a broader range of pests.

## 4. Materials and Methods

### 4.1. Collection and Preparation of Plant Sample

The *C. tora* plant was collected from the Botanical Garden of the Department of Plant Biology, Federal University Dutse, Nigeria. Using a paper envelope, this plant sample was sealed and labelled at the collection point, then transported to the laboratory for further processing. While being transferred to the lab, the leaf sample was carefully separated from the rest of the plant, followed by consecutive rinsing under running tap water, then with 1% sodium hypochlorite to get rid of contaminants from the leaf’s surface. Then, the cleaned sample was allowed to dry at room temperature to preserve the nature of the phytochemical composition. The leaf sample was allowed to dry completely until a constant weight was obtained, determined with a Sci-Chem (CLS 501) weighing balance. The dried sample was pulverised into fine powder using a sterile mortar and pestle, sieved and sealed in a sterile container and kept at 37 °C before use.

### 4.2. Phytochemical Extraction and Identification

Following standard procedures, the processed sample was extracted with methanol (1:10 *w*/*v*) under sonication (40 kHz, 30 °C, 45 min). The extract was reconstituted in HPLC-grade methanol (1 mg/mL), filtered through Whatman No. 1 paper (Whatman, Maidstone, UK), and concentrated under lowered pressure at 40 °C. For the phytocompound identification, an Agilent Technologies 5977 MSD GC-MS system (Agilent Technologies, Santa Clara, CA, USA) with a DB-5MS capillary column (30 m × 0.25 mm × 0.25 μm) was used to analyse the obtained methanolic extract of *C. tora* leaves. Using helium as the carrier gas (1.0 mL/min flow rate) and a split ratio of 10:1, chromatographic separation was attained. Starting at 50 °C (kept for 2 min), the temperature programme ran to 300 °C at 10 °C/min and held for 10 min. Injector and ion source temperatures were set to 250 °C and 230 °C, respectively. Scanning *m*/*z* 50–600, mass spectra were obtained in electron ionisation (EI) mode at 70 eV. Using the NIST 2020 collection, compounds were found with a similarity index threshold of over 80% to guarantee confidence in matches. Using a homologous series of n-alkanes (C8–C40), retention indices (RI) were computed and compared to published values for verification. Agilent MassHunter Workstation software (v.10.0) was used to accomplish data collecting and processing.

### 4.3. Ligand Retrieval and Preparation

The identified compounds from GC-MS analysis were evaluated for duplicate compound names. The chemical information of the phytocompound was retrieved from the PubChem database (https://pubchem.ncbi.nlm.nih.gov/) through the search by name protocol, where the Structure Data File (SDF) of each compound was extracted in 3D format. The 2D format of the compounds without available 3D structure was also extracted from the database and converted to 3D SDF form using Open Babel (openbabel-3-1-1) tool [36]. These compounds were saved and used as ligands in the docking analysis.

### 4.4. Protein Preparation

Three target proteins each from *T. absoluta* (Ryanodine receptor, Sodium Channel Protein and Kruppel-like protein 1) and *A. solani* (Effector Protein AsCEP50, Polygalacturonase and Mitogen-activated protein kinase HOG1) were used as receptors in this study. The 3D structure of proteins (Ryanodine receptor, Sodium Channel Protein, Polygalacturonase and Mitogen-activated protein kinase HOG1) with IDs A0AAU7VFH4, I3PW14, A0A2U7QRA7 and D4NW87 were retrieved from the Uniprot (https://www.uniprot.org/) protein database in Protein Data Bank (PDB) format. Because the 3D structures of Kruppel-like protein 1 and effector protein AsCEP50 were not found, the structures of these proteins were predicted according to their respective protein sequences. The protein sequence of Kruppel-like protein 1 (WRO29221.1) and the nucleotide gene sequence of Effector Protein AsCEP50 (OM735615.1) were retrieved from NCBI (https://www.ncbi.nlm.nih.gov/). Prior to structure modelling, the nucleotide sequence of the effector protein AsCEP50 was translated to a protein FASTA sequence, and the Open Reading Frame (ORF) was using the Swiss-Prot Expert Protein Analysis System (ExPASy) [37,38].

The obtained ORF was further compared and confirmed in the NCBI ORF finder viewer (https://www.ncbi.nlm.nih.gov/orffinder/20210817/) accessed on 27 February 2025. Afterwards, protein structure homology modelling of these proteins was predicted using the Swiss-Model server (https://swissmodel.expasy.org/interactive) accessed on 15 March 2025, and the quality and validity of the predicted proteins were assessed based on sequence coverage and similarity, QMEANDisCo Global, GMQE Score and Ramachandran Plot Statistics [39,40]. Protein Quality Predictor (ProQ3) was used to further validate the predicted protein structures [41].

### 4.5. Molecular Docking Analysis

Molecular docking was used to ascertain the interaction between the identified *C. tora* bioactive phytochemicals and the target proteins involved with *T*. *absoluta* and *A. solani*. Before docking, proteins were processed with PyMOL Molecular Graphics System Version 3.1.6.1 (Schrödinger, LLC, New York, NY, USA) by deleting water molecules, heteroatoms, and co-crystallised ligands which might interfere with the ligand-binding site. Polar hydrogens were added, and the models were output as PDB files for docking. The phytochemical ligands were prepared using Python Prescription (PyRx) Version 0.8, which integrates virtual screening software packages AutoDock Vina Version 1.2.7 (The Scripps Research Institute, La Jolla, CA, USA) and Open Babel Version 2.4.1 (Graphical User Interface), Open Babel Development Team (http://openbabel.org). for energy optimisation and file format conversion [42]. Ligands were subjected to energy minimisation to attain stable conformations and converted to the PDBQT format required for docking [43]. Blind docking simulations were performed in PyRx using the scoring function of AutoDock Vina to predict binding affinities and to generate the best binding poses. Protein–ligand interactions following docking analysis were performed using BIOVIA Discovery Studio Visualiser (Dassault Systèmes, 2025 Client (Dassault Systèmes, Vélizy-Villacoublay, France) for the shedding of binding interactions [44], such as hydrogen bonds, hydrophobic contact and π–π stacking interactions, allowing for an identification of critical residues interacting with the ligand and the type of interactions contributing to the binding affinity. MD simulations were run on complexes with the greatest binding affinities.

### 4.6. Molecular Dynamic Simulation (MDS) Analysis

MD simulations were performed on identified top phytochemicals against *T. absoluta* proteins, namely, Kruppel-like protein 1, ryanodine receptor, and sodium channel protein; and *A. solani* proteins, namely, effector protein, endopolygalacturonase, and mitogen-activated protein kinase. Specifically, in the case of *T. absoluta,* the complexes of Kruppel-like protein 1 with BHT and 4,7,10,13,16,19-docosahexaenoic acid methyl ester, the complexes of ryanodine receptor with squalene and butylated hydroxytoluene (BHT), and the complexes of sodium channel protein with BHT and squalene were selected for the extensive MD simulations. While, in the case of *A. solani,* the complexes of effector protein with BHT and squalene, the complexes of endopolygalacturonase with 4,7,10,13,16,19-docosahexaenoic acid methyl ester, BHT, and squalene, and the complexes of mitogen-activated protein kinase with squalene and 4,7,10,13,16,19-docosahexaenoic acid methyl ester were selected for the MD simulations. The purpose of MD simulations was to obtain detailed insights into the binding affinities and gauge the possible mode of binding of the respective phytochemical at the binding site of selected proteins. The MD simulations of a 100 ns duration were performed using the Gromacs (version 2020.4) program [45]. The topology of the respective protein was constructed from the Amber ff99SB protein force field [46], while the topologies of the respective phytochemicals were parameterised using the Acpype interface using the General Atom Force Field (GAFF) [47]. The solvation of resultant complexes of protein–ligand was afforded with the TIP3P water model in a dodecahedron unit cell where the system edges were kept 10 Å away from the edges of the box [48]. The resultant systems of respective protein–ligand complexes were then neutralised with the addition of an appropriate number of sodium and chloride ions. The reliving of steric clashes and positioning of solvent molecules was achieved through the energy minimisation with a combination of the steepest descent [49] and the conjugate gradient [50] methods, where the force constant was set to 100 kJ mol^−1^ nm^−1^. The resultant systems were then sequentially subjected to the temperature and pressure equilibrations (NVT and NPT) using the constant temperature of 300 K (NVT) conditions using the modified Berendsen thermostat [51] and then at constant pressure of 1 atm (NPT) conditions using the Berendsen barostat [52]. The unrestrained production phase 100 ns MD simulations were then performed where the temperature and pressure conditions were maintained from the modified Berendensen thermostat and the Parrinello-Rahman barostat [53], respectively. The covalent bonds were restrained with the LINCS algorithm [54] and the electrostatic energies at the distance of 12 Å were computed with the Particle Mesh Ewald (PME) method [55]. The resultant trajectories were first treated for the periodic boundary conditions and then analysed for the root mean square deviations (RMSD) in the backbone atoms, the RMSD in ligand atoms, the root mean square fluctuation (RMSF) in the side chain atoms, and the radius of gyration. The solvent-accessible surface areas (*SASA*) were calculated for respective proteins, ligands, and protein-ligand complexes. From these *SASA* measurements the buried solvent-accessible surface area (*B-SASA*) was calculated from Equation (1).

(1) $$B\text{-}SASA\;\left({Å}^{2}\right)=SASA_{protein\;}+SASA_{Ligand}-SASA_{protein\;ligand\;complex}$$

The hydrogen bond analysis was performed on respective protein–ligand complexes. The further insights into the other non-bonded interactions were from the contact frequency analysis within a distance of 3.5 Å using the MDCiao [56] program and Protein–Ligand Interaction Fingerprints (ProLIF) analysis [57]. The trajectories at various time intervals were visually inspected for the non-bonded interactions. The principal component analysis (PCA) [58] on the trajectories was carried out to analyse the metastable conformations from the first two principal components and the resultant Gibbs free-energy landscapes [59]. In the PCA the covariance matrix was constructed and then diagonalised to obtain two principal components for the respective protein–ligand complexes employing the backbone atoms of proteins and atoms of bound ligands. The binding affinity was gauged from the MM-GBSA calculation from the Molecular Mechanics General Born surface area and surface area solvation (MM/GBSA) calculation [60]. In the MM-GBSA calculations, trajectories from 50 to 100 ns at each 50 ps time step were subjected to the calculation to obtain the binding free energies (ΔG_binding_ kcal/mol).

### 4.7. Toxicity Analysis

The toxicity profiles of the top compounds (squalene, 4,7,10,13,16,19-docosahexaenoic acid methyl ester (DHAME), and butylated hydroxytoluene (BHT)), with pesticidal potentials, were predicted using ProTox-3.0, a computational tool combining molecular descriptors and machine-learning models estimating acute toxicity (LD50), toxicity classes, and interactions with biological targets (e.g., nuclear receptors, enzymes, and stress pathways) [61]. Toxicity profiling took into account the significant parameters, particularly hepatotoxicity, carcinogenicity, mutagenicity, mitochondrial membrane disruption, blood–brain barrier (BBB) penetration, and cytochrome P450 (CYP) suppression. The threshold for active probability was set at ≥0.60. Additionally, to evaluate the potential environmental impact and safety profile of the identified lead phytocompounds, another in silico ecological toxicity prediction was performed using the Ecological Structure Activity Relationships (ECOSAR) predictive model (v2.0). This screening-level quantitative structure–activity relationship (QSAR) tool, developed by the U.S. Environmental Protection Agency (U.S. EPA), estimates acute and chronic aquatic toxicities based on a chemical structure and physicochemical properties. The Simplified Molecular Input Line Entry System (SMILES) strings for each compound were retrieved from the PubChem database and used as input into the EPI Suite™ software (v4.11). Toxicity endpoints for three representative aquatic trophic levels—fish (96 h LC50), Daphnia (48 h LC50), and green algae (96 h EC50)—were predicted and reported in mg/L. Predicted physicochemical parameters, notably the octanol–water partition coefficient (log K_ow_), water solubility (mg L^−1^), and bioconcentration factors (L/kg wet-wt), were also obtained.

## 5. Conclusions

This study demonstrates that *Cassia tora* harbours bioactive compounds with significant pesticidal potential against *T. absoluta* and *A. solani*. Molecular dynamics and MM-GBSA analyses identified squalene and DHAME as the most potent ligands, forming energetically stable and persistent hydrophobic interactions with critical insect and fungal proteins. These findings highlight their dual functionality as natural insecticidal and antifungal agents capable of targeting neuromuscular and pathogenic signalling pathways. Due to their stable binding, predicted safety, and plant-based nature, squalene and DHAME are promising candidates for developing sustainable and eco-friendly biopesticides. Using squalene and DHAME could significantly reduce reliance on chemical pesticides and improve integrated pest management in agricultural systems facing climate stress, especially if validated through further in vitro or in vivo studies.

## Figures and Tables

**Table 1:** Phyto-compounds Identified from *C. tora* Plant Extract by GC-MS Analysis and Used for Molecular Docking Analysis as Potential Inhibitors Against the Target Proteins.

PK	RT	Area (%)	Library/ID	CID	Molecular Weight	Molecular Formula
**1**	13.6387	0.2004	Butylated Hydroxytoluene	31404	220.35	C_15_H_24_O
**2**	15.4266	0.192	5-Octadecene, (E)-	5364598	252.5	C_18_H_36_
**3**	18.4259	0.9165	3-Butynylbenzene	123360	130.19	C_10_H_10_
**4**	19.9252	0.3641	1-Octadecene	8217	252.5	C_18_H_36_
**5**	22.7844	5.2233	Pentadecanoic acid, 14-methyl-, methyl ester	21205	270.5	C_17_H_34_O_2_
**6**	23.5932	0.6823	Dibutyl phthalate	3026	278.34	C_16_H_22_O_4_
**7**	24.0434	0.5886	Trichloroacetic acid, pentadecyl ester	522535	373.8	C_17_H_31_Cl_3_O_2_
**8**	24.1261	1.1888	Hexadecanoic acid, ethyl ester	5284421	294.5	C_19_H_34_O_2_
**9**	26.0593	6.5583	9,12-Octadecadienoic acid, methyl ester	3931	280.4	C_18_H_32_O_2_
**10**	26.1911	19.663	9-Octadecenoic acid (Z)-, methyl ester	445639	282.5	C_18_H_34_O_2_
**11**	26.6748	5.0828	Methyl stearate	8201	298.5	C_19_H_38_O_2_
**12**	27.0888	0.5551	Methyl 8,11,14,17-eicosatetraenoate	14122970	318.5	C_21_H_34_O_2_
**13**	27.2836	0.5665	Linoleic acid ethyl ester	5282184	308.5	C_20_H_36_O_2_
**14**	27.393	2.8875	(E)-9-Octadecenoic acid ethyl ester	5364430	310.5	C_20_H_38_O_2_
**15**	27.5007	0.6396	Ethyl Oleate	5363269	310.5	C_20_H_38_O_2_
**16**	27.8197	0.4702	1-Docosene	74138	308.6	C_22_H_44_
**17**	27.8931	1.2984	Octadecanoic acid, ethyl ester	8122	312.5	C_20_H_40_O_2_
**18**	29.0123	0.2513	Methyl 9,12-heptadecadienoate	14162504	280.4	C_18_H_32_O_2_
**19**	29.108	3.3506	n-Propyl 11-octadecenoate	87131822	324.5	C_21_H_40_O_2_
**20**	29.5913	0.9662	Octadecanoic acid, propyl ester	77190	326.6	C_21_H_42_O_2_
**21**	29.7735	0.4278	cis-11-Eicosenoic acid	5282768	310.5	C_20_H_38_O_2_
**22**	30.1177	0.3479	6-Butyl-1,4-cycloheptadiene	556470	150.26	C_11_H_18_
**23**	30.2541	13.5929	cis-5,8,11,14,17-Eicosapentaenoic acid	446284	302.5	C_20_H_30_O_2_
**24**	30.6097	0.7136	7,10,13-Hexadecatrienoic acid, methyl ester	556196	264.4	C_17_H_28_O_2_
**25**	31.0307	0.2023	Oleic Acid	445639		
**26**	31.2839	0.3775	Heptadecyl heptafluorobutyrate	545577	452.5	C_21_H_35_F_7_O_2_
**27**	31.3444	0.439	Ethanol, 2-(tetradecyloxy)-	16491	258.44	C_16_H_34_O_2_
**28**	32.4882	0.2459	cis-Vaccenic acid	5282761	282.5	C_18_H_34_O_2_
**29**	33.1772	0.7821	Erucic acid	5281116	338.6	C_22_H_42_O_2_
**30**	33.316	15.2724	4,7,10,13,16,19-Docosahexaenoic acid, methyl ester, (all-Z)-	5353594	328.5	C_22_H_32_O_2_
**31**	33.5667	4.5695	Methyl 6,9,12,15,18-heneicosapentaenoate	72733997	330.5	C_22_H_34_O_2_
**32**	33.8723	1.705	Diethyl Phthalate	6781	222.24	C_12_H_14_O_4_
**33**	34.0849	2.8543	3-Eicosene, (E)-	5365051	280.5	C_20_H_40_
**34**	34.296	0.5693	9-Eicosenoic acid, (Z)-	5282767	310.5	C_20_H_38_O_2_
**35**	34.3682	0.1159	i-Propyl 9-tetradecenoate	56936038	268.4	C_17_H_32_O_2_
**36**	34.6783	0.2119	13-Octadecenal, (Z)-	5364497	266.5	C_18_H_34_O
**37**	37.8522	1.3136	Squalene	638072	410.7	C_30_H_50_
**38**	38.4314	−0.1833	2-Methyl-Z,Z-3,13-octadecadienol	5364412	280.5	C_19_H_36_O

**Table 2:** MM-GBSA Analysis for *T. absoluta* Complexes.

*T. absolata* Protein Ligand Complexes	Energy Component (kcal/mol)								
	ENTROPY (−TΔS)	ΔVDWAALS	ΔEEL	ΔEGB	ΔESURF	ΔGGAS	ΔGSOLV	ΔTOTAL	ΔG Binding
**Complex of Kruppel-like protein 1**									
Butylated hydroxytoluene	13.23 (0.0)	−5.18 (1.68)	−0.03 (0.05)	0.53 (0.32)	−0.67 (0.38)	−5.22 (1.68)	−0.13 (0.50)	−5.35 (1.75)	7.88 (7.95)
4,7,10,13,16,19-docosahexaenoic acid, methyl ester	40.80 0.05	−40.22 0.15	−3.34 0.32	6.63 0.17	−6.27 0.10	−43.56 0.36	0.35 0.19	−43.21 0.41	−2.40 ± 6.07
**Ryanodine receptor**									
Squalene	16.08 0.05	−51.08 1.44	−0.45 0.17	4.20 0.03	−6.84 0.18	−51.53 1.45	−2.64 0.18	−54.17 1.46	−38.09 ± 4.99
Butylated hydroxytoluene	14.28 0.05	−19.59 0.31	−0.32 0.06	1.74 0.16	−2.62 0.12	−19.91 0.32	−0.88 0.20	−20.79 0.38	−6.51 ± 6.68
**Sodium Channel Protein**									
Butylated hydroxytoluene	19.24 0.04	−19.24 0.57	−0.45 0.02	1.70 0.06	−2.49 0.06	−19.68 0.57	−0.79 0.08	−20.47 0.57	−1.23 ± 7.26
Squalene	32.07 0.05	−37.61 0.22	−0.17 0.14	2.53 0.02	−5.33 0.13	−37.78 0.26	−2.81 0.13	−40.58 0.29	−8.52 ± 8.56

**Table 3:** MM-GBSA Analysis for *A. solani* Complexes.

*A. solani* Protein Ligand Complexes	Energy Component (kcal/mol)								
	ENTROPY (−TΔS)	ΔVDWAALS	ΔEEL	ΔEGB	ΔESURF	ΔGGAS	ΔGSOLV	ΔTOTAL	ΔG Binding
**Effector Protein AsCEP50**									
Butylated hydroxytoluene	10.86 (0.05)	−28.21 (1.51)	−0.70 (0.03)	2.82 (0.01)	−3.60 (0.09)	−28.90 (1.51)	−0.78 (0.09)	−29.68 (1.52)	−18.82 (3.73)
Squalene	27.49 14.95	−41.11 1.70	−0.17 0.17	3.90 0.05	−5.71 0.15	−41.28 1.71	−1.81 0.15	−43.08 1.71	−15.59 ± 16.29
**Polygalacturonase (endopolygalacturonase)**									
4,7,10,13,16,19-docosahexaenoic acid, methyl ester	28.23 0.05	−31.29 0.01	−0.92 0.29	3.30 0.02	−5.24 0.08	−32.21 0.29	−1.94 0.09	−34.15 0.30	−5.92 ± 6.86
Butylated hydroxytoluene	24.21 0.21	−28.37 0.59	−0.22 0.06	2.23 0.03	−3.64 0.05	−28.58 0.59	−1.42 0.06	−30.00 0.60	−5.79 ± 3.86
Squalene	29.93 0.05	−38.32 2.62	−0.17 0.14	2.49 0.04	−5.59 0.58	−38.50 2.62	−3.10 0.58	−41.60 2.69	−11.67 ± 12.14
**Mitogen-activated protein kinase HOG1**									
Squalene	19.05 0.05	−67.01 1.78	−0.35 0.13	4.61 0.04	−9.11 0.14	−67.36 1.78	−4.50 0.14	−71.86 1.79	−52.81 ± 5.82
4,7,10,13,16,19-docosahexaenoic acid, methyl ester	13.66 0.05	−42.91 0.27	−2.32 0.51	5.49 0.69	−6.57 0.04	−45.23 0.58	−1.08 0.69	−46.31 0.90	−32.65 ± 4.18

ΔVDWAALS: van der Waals energy; ΔEEL: Electrostatic energies; ΔEGB: Polar solvation energy; ΔESURF: Nonpolar solvation energy; ΔGGAS = ΔVDWAALS + ΔEEL; ΔGSOLV = ΔEGB+ ΔESURF; ΔTOTAL = ΔGSOLV +ΔGGAS; ΔG binding = ΔTOTAL − TΔS (Standard deviations are given in parentheses).

**Table 4:** Toxicity Profile of the Top Compounds from *C. tora* with Biopesticidal Potential.

Target	Squalene		Butylated Hydroxytoluene		DHAME	
	Prediction	Probability	Prediction	Probability	Prediction	Probability
Hepatotoxicity	Inactive	0.79	Inactive	0.78	Active	0.69
Neurotoxicity	Inactive	0.65	Inactive	0.50	Active	0.87
Nephrotoxicity	Inactive	0.88	Inactive	0.84	Inactive	0.90
Respiratory toxicity	Inactive	0.59	Inactive	0.93	Active	0.98
Cardiotoxicity	Inactive	0.79	Inactive	0.98	Inactive	0.77
Carcinogenicity	Inactive	0.76	Inactive	0.52	Inactive	0.62
Immunotoxicity	Inactive	0.99	Inactive	0.98	Active	0.96
Mutagenicity	Inactive	0.98	Inactive	0.99	Inactive	0.97
Cytotoxicity	Inactive	0.81	Inactive	0.91	Inactive	0.93
BBB-barrier	Active	0.97	Active	0.94	Inactive	1
Ecotoxicity	Active	0.62	Active	0.63	Active	0.73
Clinical toxicity	Inactive	0.77	Inactive	0.66	Inactive	0.56
Nutritional toxicity	Inactive	0.83	Inactive	0.96	Inactive	0.74
Aryl hydrocarbon Receptor (AhR)	Inactive	0.99	Inactive	1.0	Inactive	0.97
Androgen Receptor (AR)	Inactive	0.98	Inactive	1.0	Inactive	0.99
Androgen Receptor Ligand Binding Domain (AR-LBD)	Inactive	0.91	Inactive	1.0	Inactive	0.99
Aromatase	Inactive	1.0	Inactive	0.99	Active	1
Estrogen Receptor Alpha (ER)	Inactive	0.81	Inactive	1.0	Active	0.99
Estrogen Receptor Ligand Binding Domain (ER-LBD)	Inactive	0.89	Inactive	1.0	Active	1
Peroxisome Proliferator Activated Receptor Gamma (PPAR-Gamma)	Inactive	1.0	Inactive	1.0	Inactive	0.99
Nuclear factor (erythroid-derived 2)-like 2/antioxidant responsive element (nrf2/ARE)	Active	0.60	Inactive	1.0	Inactive	0.88
Heat shock factor response element (HSE)	Active	0.60	Inactive	1.0	Inactive	0.88
Mitochondrial Membrane Potential (MMP)	Inactive	0.99	Active	0.96	Inactive	0.70
Phosphoprotein (Tumour Supressor) p53	Inactive	1.0	Inactive	0.99	Inactive	0.96
ATPase family AAA domain-containing protein 5 (ATAD5)	Inactive	1.0	Inactive	1.0	Inactive	0.99
Thyroid hormone receptor alpha (THRα)	Inactive	0.90	Inactive	0.90	Inactive	0.55
Thyroid hormone receptor beta (THRβ)	Inactive	0.78	Inactive	0.78	Inactive	0.75
Transtyretrin (TTR)	Inactive	0.97	Inactive	0.97	Inactive	0.75
Ryanodine receptor (RYR)	Inactive	0.98	Inactive	0.98	Inactive	0.93
GABA receptor (GABAR)	Inactive	0.96	Inactive	0.96	Inactive	0.76
Glutamate N-methyl-D-aspartate receptor (NMDAR)	Inactive	0.92	Inactive	0.92	Inactive	0.89
alpha-amino-3-hydroxy-5-methyl-4-isoxazolepropionate receptor (AMPAR)	Inactive	0.97	Inactive	0.97	Inactive	1
Kainate receptor (KAR)	Inactive	0.99	Inactive	0.99	Inactive	1
Achetylcholinesterase (AchE)	Inactive	0.79	Inactive	0.78	Active	0.60
Constitutive androstane receptor (CAR)	Inactive	0.98	Inactive	0.98	Inactive	0.99
Pregnane X receptor (PXR)	Inactive	0.92	Inactive	0.92	Inactive	0.69
NADH-quinone oxidoreductase (NADHOX)	Inactive	0.97	Inactive	0.97	Inactive	0.82
Voltage gated sodium channel (VGSC)	Inactive	0.95	Inactive	0.95	Inactive	0.64
Na+/I− symporter (NIS)	Inactive	0.98	Inactive	0.98	Inactive	0.79
Cytochrome CYP1A2	Inactive	0.94	Inactive	0.93	Inactive	0.76
Cytochrome CYP2C19	Inactive	0.94	Inactive	0.53	Inactive	0.87
Cytochrome CYP2C9	Active	0.67	Active	0.70	Active	0.56
Cytochrome CYP2D6	Inactive	0.77	Inactive	0.90	Inactive	0.63
Cytochrome CYP3A4	Inactive	0.99	Inactive	0.98	Active	0.71
Cytochrome CYP2E1	Inactive	0.96	Inactive	1.0	Inactive	0.98

**Table 5:** Ecological Structure Activity Relationships Predictions of the Potential *C. tora* Compounds Against *T. absoluta* and *A. solani*.

Parameter Squalene DHAME BHT			
CAS	000111-02-4	301-01-9	000128-37-0
Molecular Weight	410.719	342.516	220.351
Molecular Formula	C_30_H_50_	C_23_H_34_O_2_	C_15_H_24_O
Predicted log K_ow_	14.122	8.905	5.029
Experimental Log K_ow_	-	-	5.1
Predicted Melting Point	58.848	98.179	83.013
Predicted Boiling Point	452.899	416.847	296.493
Experimental Boiling Point	-	-	265
Predicted Vapour Pressure (mmHg at 25 °C)	1.14 × 10^−6^	6.28 × 10^−7^	0.002
Predicted Vapour Pressure (Pa at 25 °C)	1.52 × 10^−4^	8.37 × 10^−5^	0.236
Predicted Sub-cooled Vapour Pressure (mmHg at 25 °C)	1.14 × 10^−6^	3.23 × 10^−6^	0.005
Predicted Sub-cooled Vapour Pressure (Pa at 25 °C)	1.52 × 10^−4^	4.30 × 10^−4^	0.641
Experimental Vapour Pressure (mmHg at 25 °C)	-	-	0.005
Predicted HLC, VP/WSOL Method (atm-m^3^/mol at 25 °C)	925.962	0.002	0.002
Predicted HLC, Bond Method (atm-m^3^/mol at 25 °C)	341.538	0.023	4.12 × 10^−6^
Predicted HLC, Group Method (atm-m^3^/mol at 25 °C)	2.034	3.88 × 10^−5^	3.38 × 10^−6^
Predicted Log Kaw	1.92	−2.80 × 10^0^	−9.92 × 10^−1^
Predicted Log Koa	12.202	11.705	6.092
Predicted Water Solubility, WSK_ow_ (mg/L)	6.65 × 10^−10^	1.71 × 10^−4^	5.748
Predicted Water Solubility, Water NT (mg/L)	4.11 × 10^−7^	0.001	10.351
Experimental Water Solubility (mg/L)	-	-	0.6
Base-Catalysed Rate Constant (L/mol-s at 25 °C)	-	0.051	-
Hydrolysis Half-Life in Days (ph7, base-catalysed)	-	1.56 × 10^3^	-
Hydrolysis Half-Life in Days (ph8, base-catalysed)	-	155.913	-
Volatilization Half-Life in Hours (Lake Model)	192.523	480.815	144.814
Volatilization Half-Life in Hours (River Model)	2.069	29.848	1.864
Dermal Absorbed Dose per Event (mg/cm^2^-event)	0.122	16.63	380.959
Dermal Absorbed Dose (mg/kg-day)	12.904	1.76 × 10^3^	4.03 × 10^4^
Bioconcentration Factor (L/kg wet-wt)	4.31	1.55 × 10^3^	645.62
Biotransformation Half Life in Days	0.00 × 10^0^	0.00 × 10^0^	0.32
Bioaccumulation Factor (L/kg wet-wt)	33.11	4.63 × 10^4^	820.48
BioWin1 (Linear Model Prediction)	0.552	0.867	0.445
BioWin2 (Non-Linear Model Prediction)	0.056	0.983	0.111
BioWin3 (Ultimate Biodegradation Timeframe)	2.292	2.881	2.269
BioWin4 (Primary Biodegradation Timeframe)	3.255	3.852	3.194
BioWin5 (MITI Linear Model Prediction)	0.446	0.46	0.253
BioWin6 (MITI Non-Linear Model Prediction)	0.146	0.192	0.042
BioWin7 (Anaerobic Model Prediction)	0.018	−1.43 × 10^−1^	−7.92 × 10^−1^
Hydrocarbon Biodegradation Half Life in Days	2.182	-	-
Predicted Log Koc (L/kg)	8.003	5.719	4.169
Predicted Photolytic/Hydroxyl Radical Reaction Rate Constant	5.34 × 10^−10^	3.46 × 10^−10^	1.83 × 10^−11^
Predicted Atmospheric Half Life in Hours (Photolytic/Hydroxyl Radical Reaction, 1.5 × 10^6^ molecules/cm^3^)	0.24	0.371	7.018
Predicted Atmospheric Half Life in Days (Photolytic/Hydroxyl Radical Reaction, 12 h days, 1.5 × 10^6^ molecules/cm^3^)	0.02	0.031	0.585
Predicted Ozone Reaction Rate Constant	2.58 × 10^−15^	1.38 × 10^−16^	-
Predicted Atmospheric Half Life in Hours (Ozone Reaction, 7 × 10^11^ molecules/cm^3^)	0.107	0.353	-
Predicted Atmospheric Half Life in Days (Ozone Reaction, 24 h days, 7 × 10^11^ molecules/cm^3^)	0.004	0.015	-
Predicted Atmospheric Half Life in Hours (Hydroxyl and Ozone Reactions)	0.074	0.181	7.018
Fugacity Model Air Mass Percentage	0.007	0.038	0.939
Fugacity Model Water Mass Percentage	1.789	6.66	8.477
Fugacity Model Soil Mass Percentage	27.442	35.572	83.349
Fugacity Model Sediment Mass Percentage	70.762	57.73	7.235
STP Model Air Mass Percentage	1.14 × 10^−5^	5.40 × 10^−5^	10.833
STP Model Sludge Mass Percentage	93.257	93.254	73.393
STP Model Biodegradation Mass Percentage	0.779	0.779	0.579
STP Model Effluent Mass Percentage	94.035	94.033	84.805

## Data Availability

The original contributions presented in this study are included in the article/Supplementary Material. Further inquiries can be directed to the corresponding author.

## Supplementary Materials

The following supporting information can be downloaded at: https://www.mdpi.com/article/10.3390/ijms27031410/s1.
