# Supplementary material for: Molecular Dynamics Insights into Cassia tora-Derived Phytochemicals as Dual Insecticidal and Antifungal Agents Against Tomato Tuta absoluta and Alternaria solani
Source: Int J Mol Sci. 2026 Jan 30;27(3):1410. doi: 10.3390/ijms27031410 (PMC12898088; doi:10.3390/ijms27031410)
Supplement: Supplementary file 1 [file ijms-27-01410-s001.zip › Figure S1-6_2DInteraction Figures.pdf]

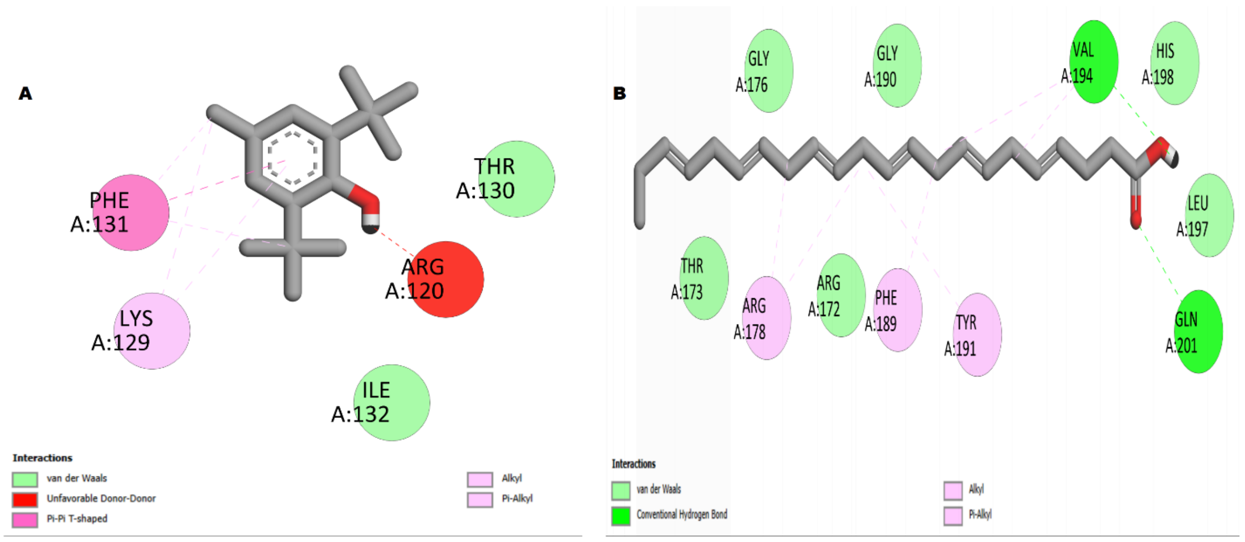

Figure S1: 2D interaction of T. absoluta Kruppel-like Protein with (A) Butylated hydroxytoluene (BHT), and (B) 4,7,10,13,16,19-docosahexaenoic acid methyl ester (DHAME).

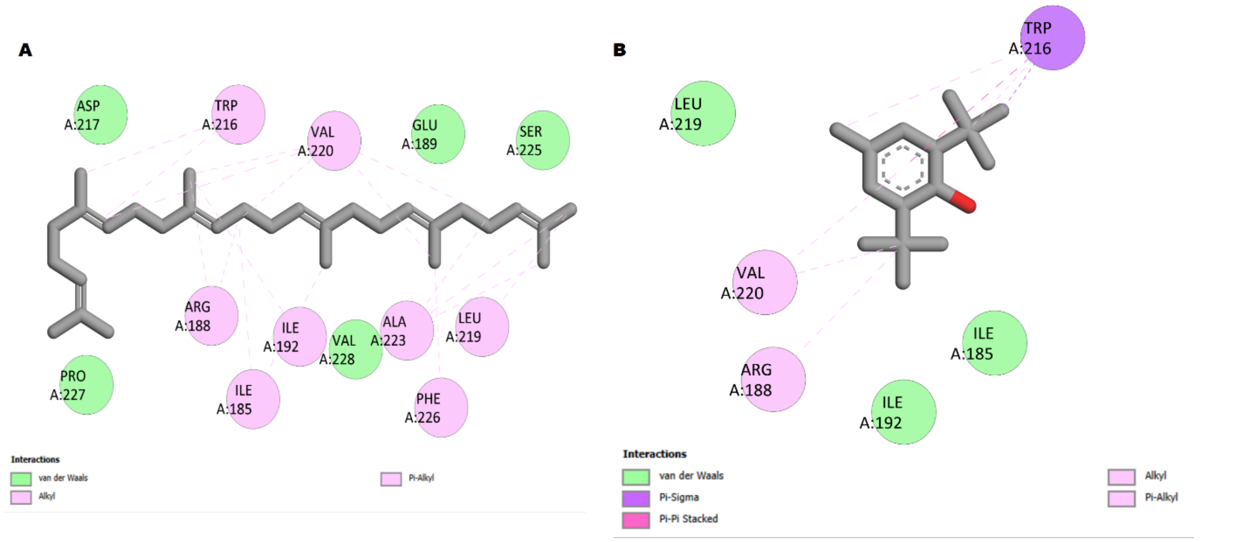

Figure S2: 2D interaction of T. absoluta Ryanodine (Ryx) Protein with (A) Squalene, and (B) Butylated hydroxytoluene (BHT).

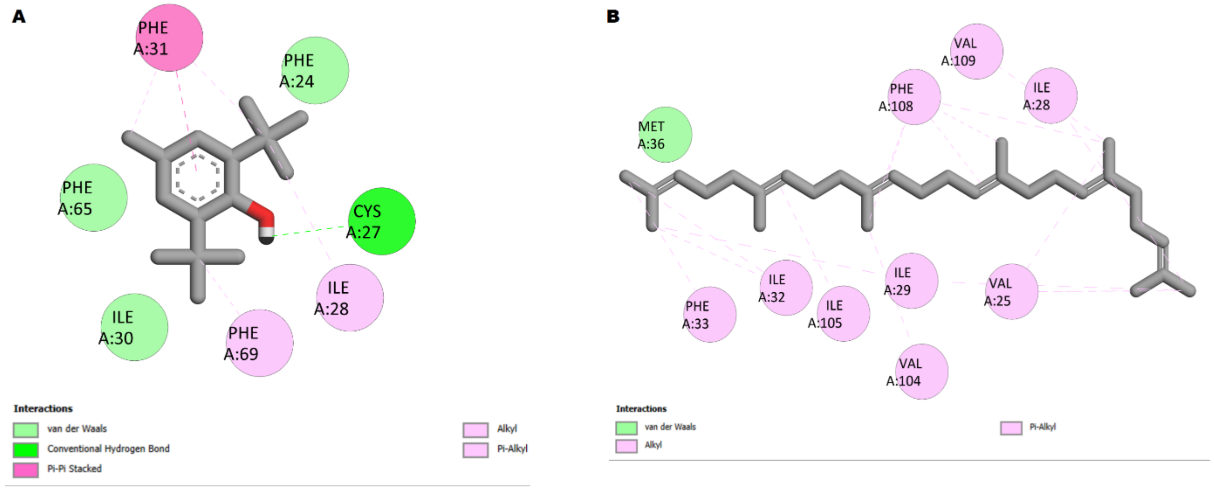

Figure S3: 2D interaction of T. absoluta Sodium Channel Protein with (A) Butylated hydroxytoluene (BHT) and (B) Squalene.

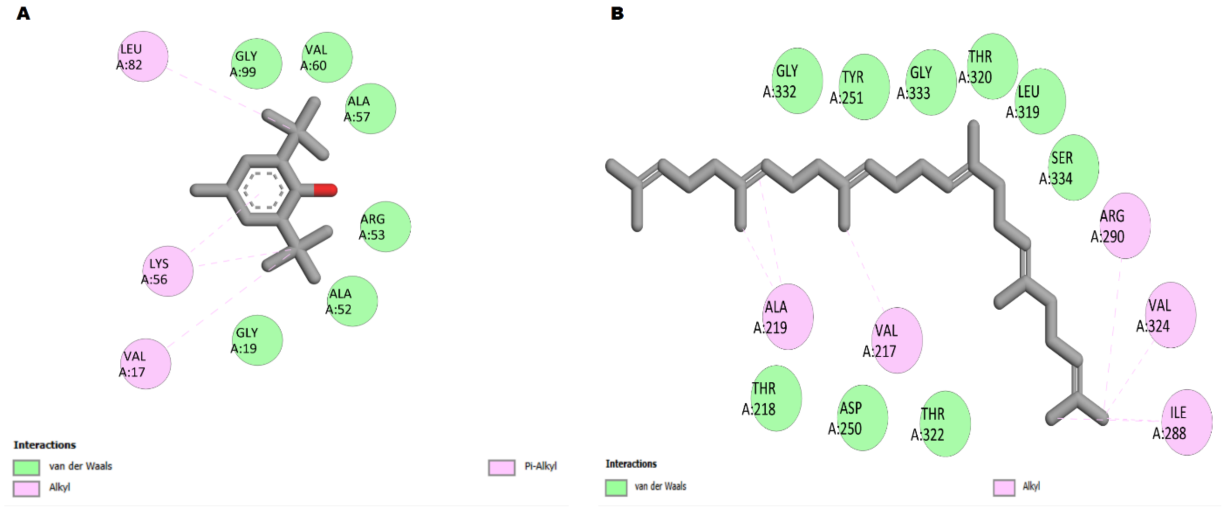

Figure S4: 2D interaction of A. solani Effector Protein (AsCEP50) with (A) Butylated hydroxytoluene (BHT) and (B) Squalene.

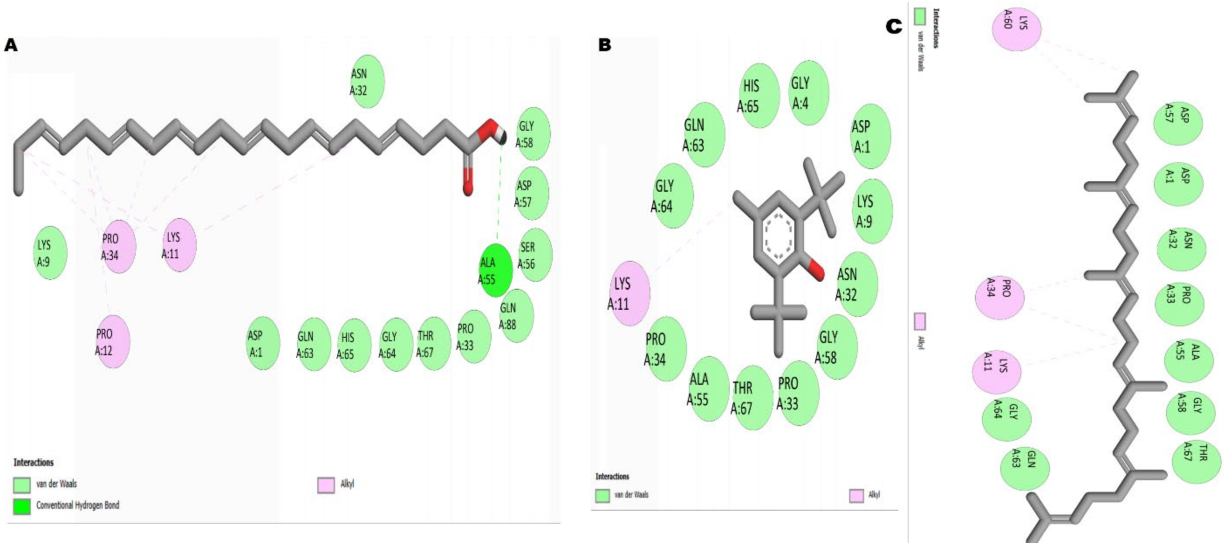

Figure S5: 2D interaction of A. solani Endopolygalacturonase with (A) 4,7,10,13,16,19-docosahexaenoic acid methyl ester (DHAME), (B) Butylated hydroxytoluene (BHT) and (C) Squalene.

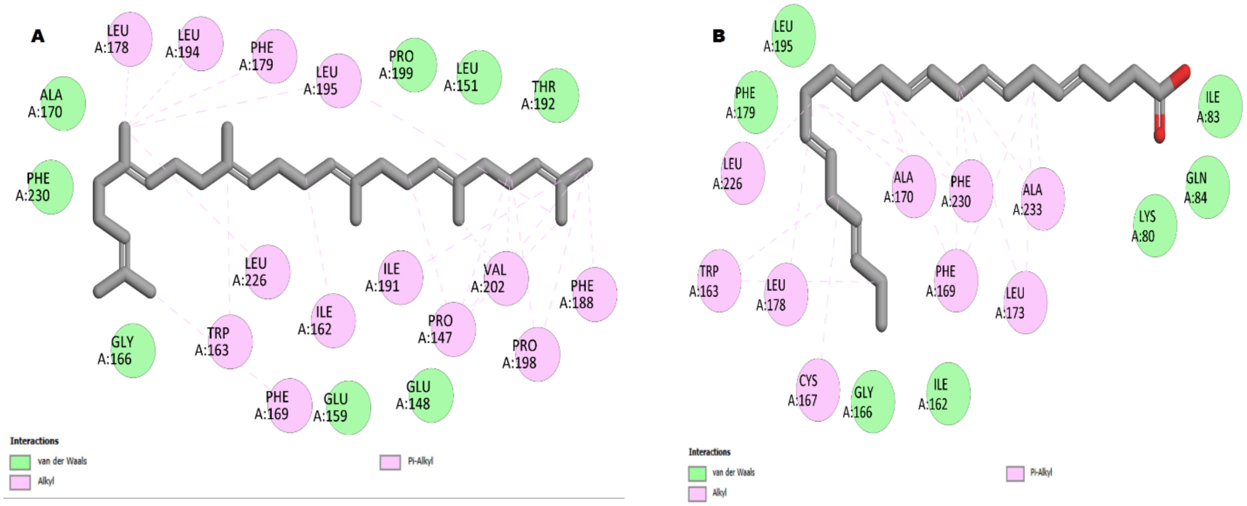

Figure S6: 2D interaction of A. solani Mitogen Activated Kinase Protein with (A) Squalene (B) 4,7,10,13,16,19-docosahexaenoic acid methyl ester (DHAME).
