# Supplementary material for: Molecular Dynamics Insights into Cassia tora-Derived Phytochemicals as Dual Insecticidal and Antifungal Agents Against Tomato Tuta absoluta and Alternaria solani
Source: Int J Mol Sci. 2026 Jan 30;27(3):1410. doi: 10.3390/ijms27031410 (PMC12898088; doi:10.3390/ijms27031410)
Supplement: Supplementary file 1 [file ijms-27-01410-s001.zip › Figure S7-14_Protein-Ligand_Fingerprinting (proLIF).pdf]

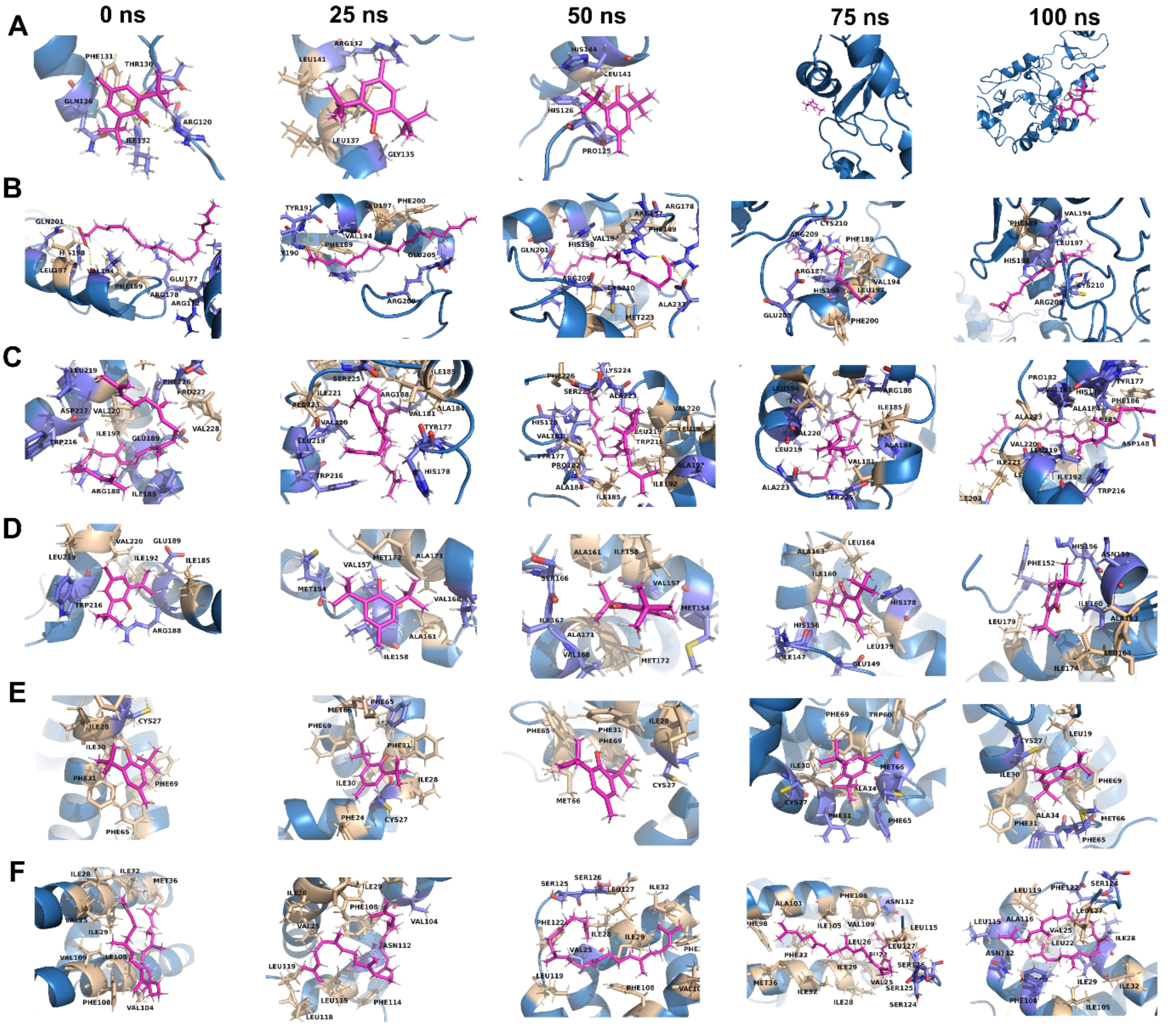

Figure S7: Trajectories at different time intervals for the T. absolata protein complexes. Complex of Kruppel-like protein 1 with A) Butylated hydroxytoluene, B) 4,7,10,13,16,19-docosahexaenoic acid, methyl ester; Complex of Ryanodine receptor with C) Squalene, D) Butylated hydroxytoluene; Complex of Sodium Channel Protein with E) Butylated hydroxytoluene, and F) Squalene; Kruppel-like protein 1 complexes with A) Butylated hydroxytoluene, B) 4,7,10,13,16,19-docosahexaenoic acid, methyl ester.

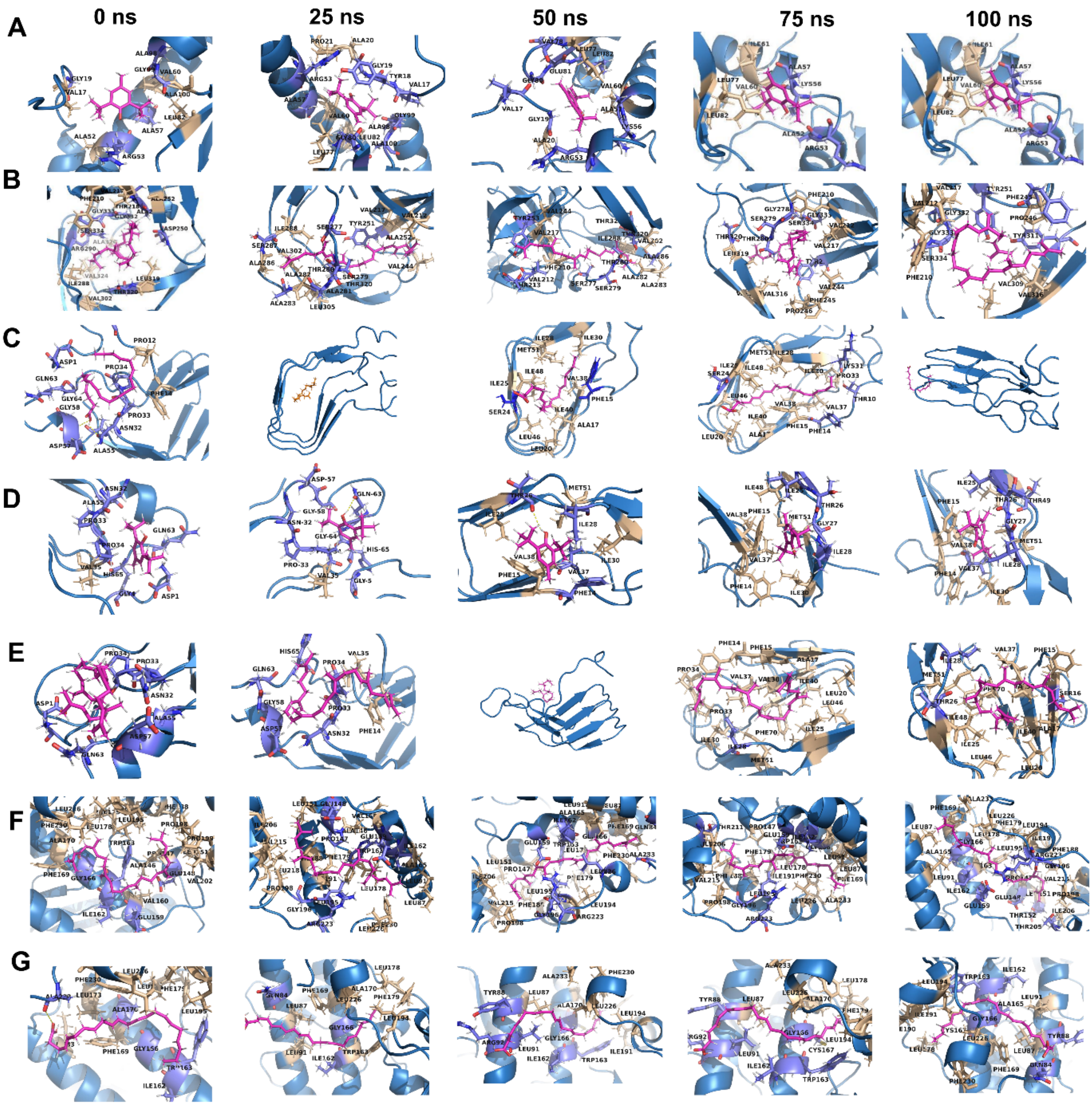

Figure S8: Trajectories at different time intervals for the A. solani protein complexes. Complex of Effector Protein AsCEP50 with A) Butylated hydroxytoluene, B) Squalene; Complexes of Polygalacturonase (endopolyglugalacturonase) with C) 4,7,10,13,16,19-docosahexaenoic acid, methyl ester, D) Butylated hydroxytoluene, E) Squalene; Complex of Mitogen-activated protein kinase HOG1 with F) Squalene, and G) 4,7,10,13,16,19-docosahexaenoic acid, methyl ester.

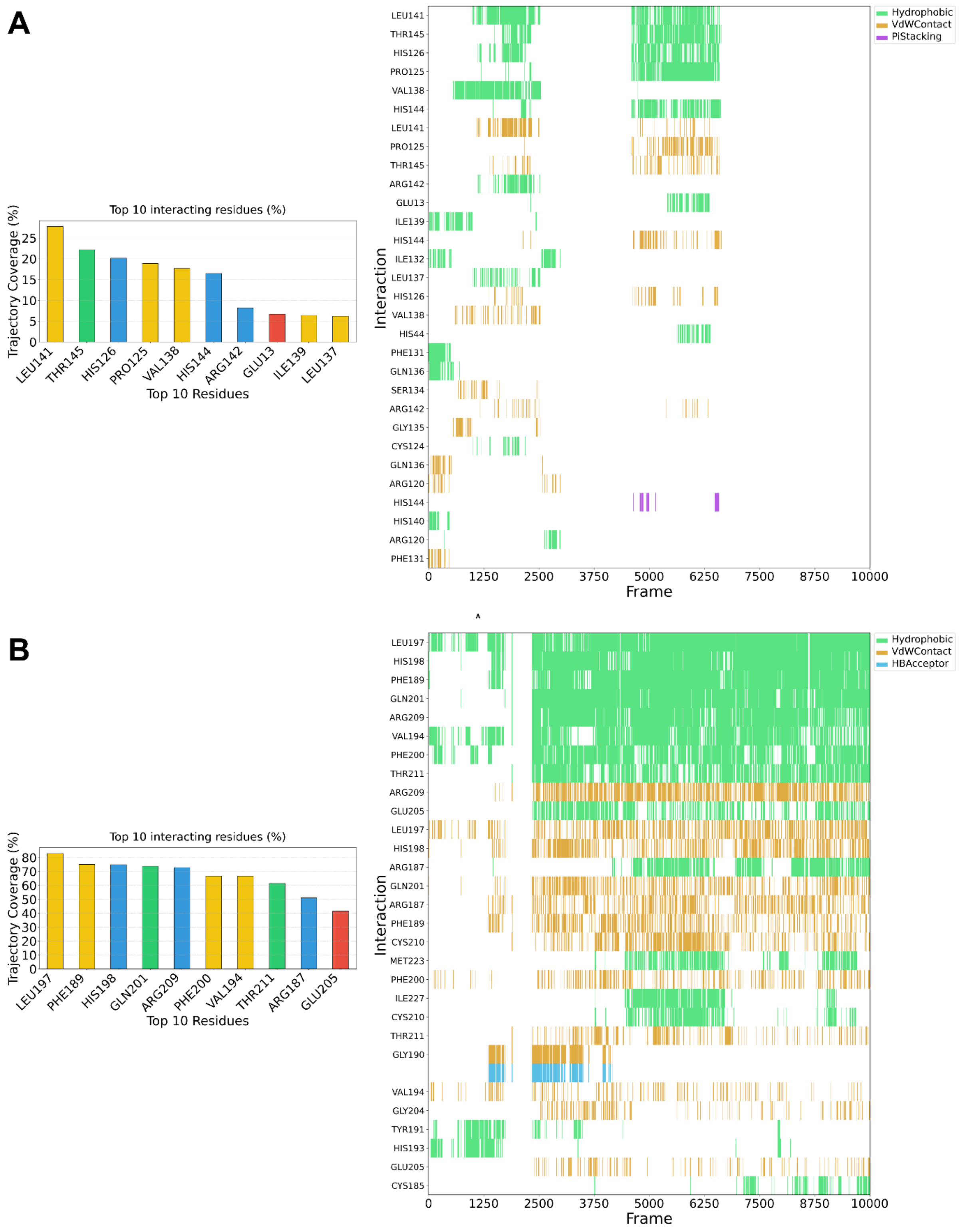

Figure S9: T. absolata Kruppel-like protein 1 complexes with A) Butylated hydroxytoluene, B) 4,7,10,13,16,19-docosahexaenoic acid, methyl ester.

A

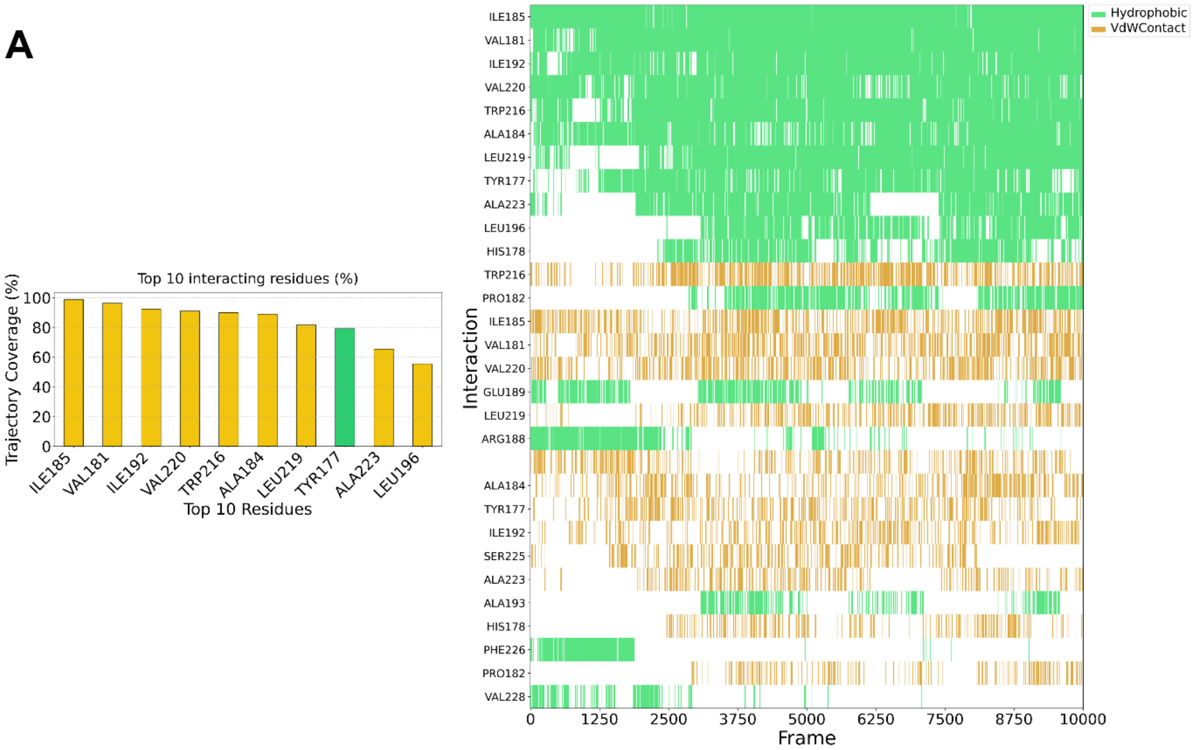

B

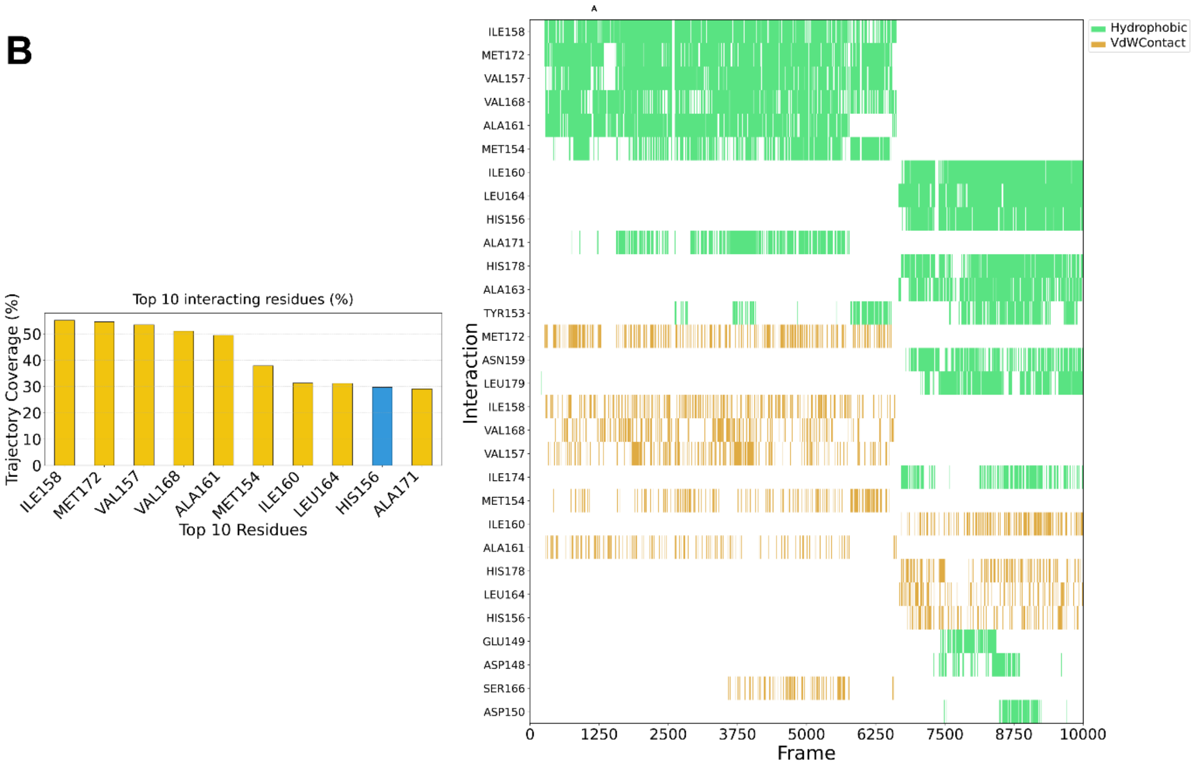

Figure S10: T. absolata Ryanodine receptor complexes with A) Squalene, and B) Butylated hydroxytoluene.

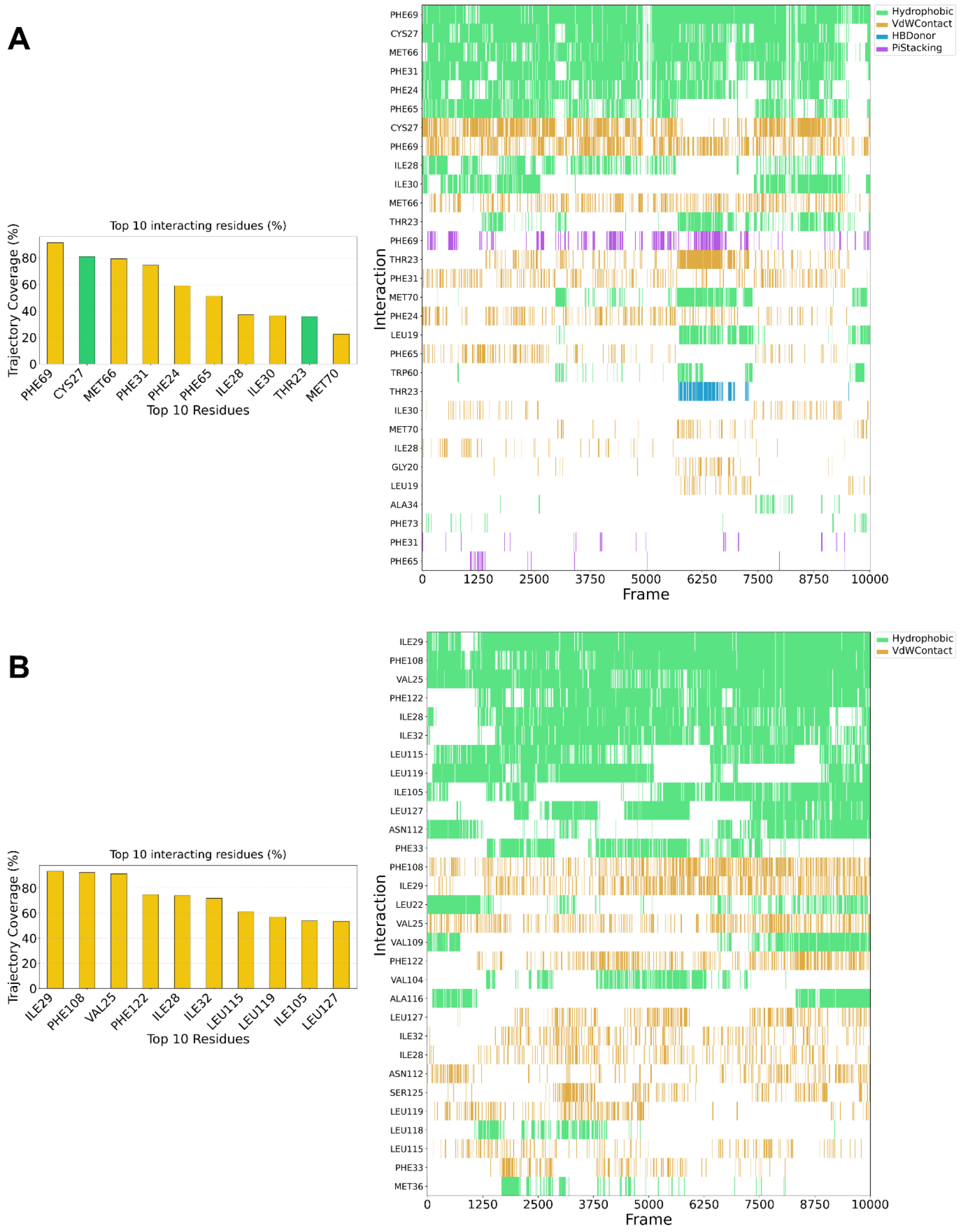

Figure S11: T. absolata Sodium Channel Protein complexes with A) Butylated hydroxytoluene, and B) Squalene.

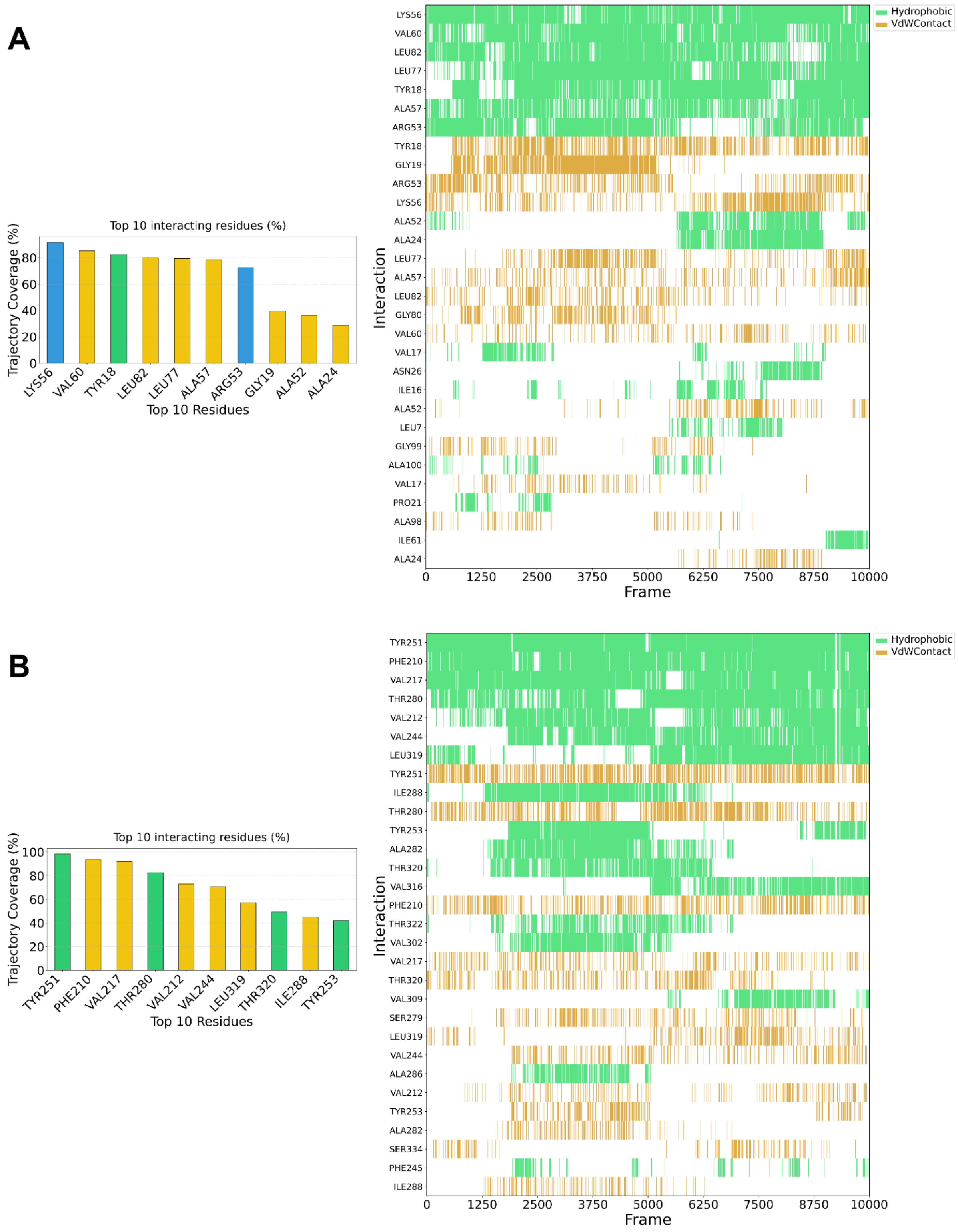

Figure S12: A. solani Complex of Effector Protein AsCEP50 with A) Butylated hydroxytoluene, B) Squalene.

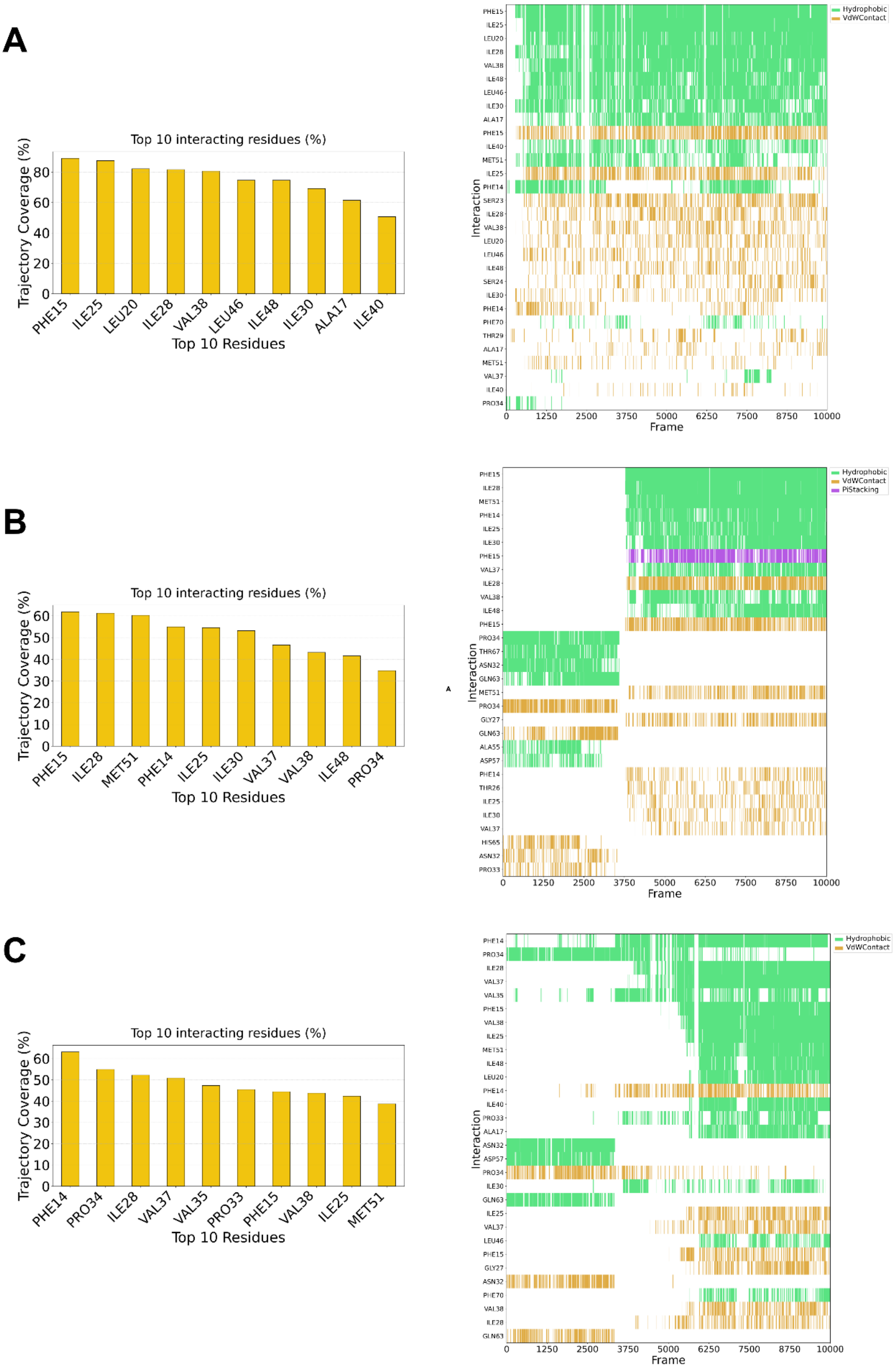

Figure S13: A. solani Complexes of Polygalacturonase (endopolyglugalacturonase) with A) 4,7,10,13,16,19-docosahexaenoic acid, methyl ester, B) Butylated hydroxytoluene, C) Squalene.

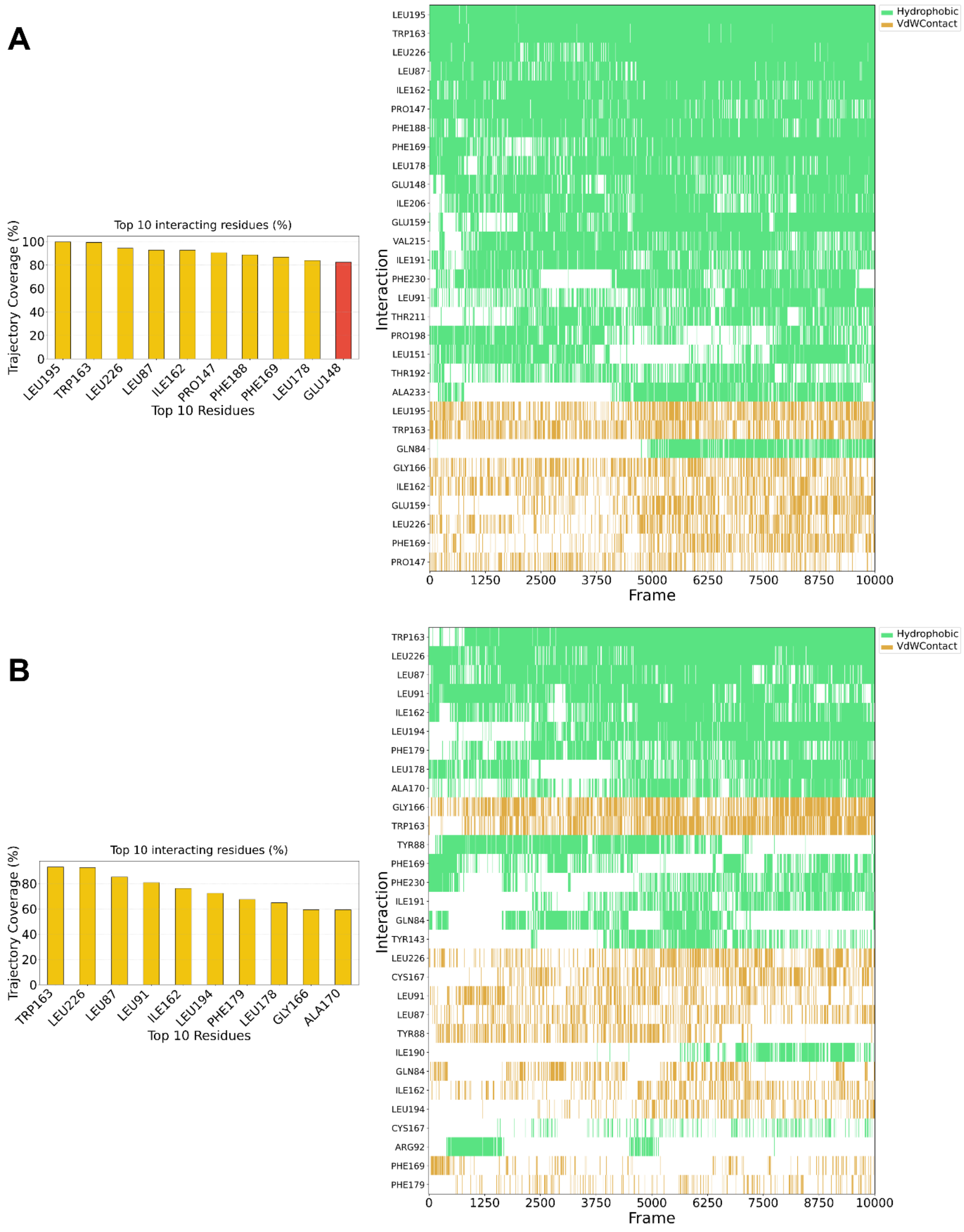

Figure S14: A. solani Complex of Mitogen-activated protein kinase HOG1 with A) Squalene and B) 4,7,10,13,16,19-docosahexaenoic acid, methyl ester.
