# Supplementary material for: Molecular Dynamics Insights into Cassia tora-Derived Phytochemicals as Dual Insecticidal and Antifungal Agents Against Tomato Tuta absoluta and Alternaria solani
Source: Int J Mol Sci. 2026 Jan 30;27(3):1410. doi: 10.3390/ijms27031410 (PMC12898088; doi:10.3390/ijms27031410)
Supplement: Supplementary file 1 [file ijms-27-01410-s001.zip › Tables S1-2_Binding Affinity (Docking Scores).pdf]

Table S1: Binding Affinities (ΔG, kcal/mol) of *Cassia tora* Bioactive Compounds Against *Tuta absoluta* Target Proteins (Kruppel Protein, Ryanodine Receptor, and Voltage-Gated Na^+ Channel)

S/No	Compounds	Binding Affinity (Kcal/mol)		
		Kruppel Protein	Ryanodine Protein	Na^+ Channel Gated Protein
1.	3-Butynylbenzene	-4.4	-4.4	-4.5
2.	Methyl 8,11,14,17-eicosatetraenoate	-3.3	-4.2	-4.7
3.	Methyl 9,12-heptadecadienoate	-3.6	-4.4	-4.3
4.	Ethanol, 2-(tetradecyloxy)-	-3.4	-3.3	-3.9
5.	Pentadecanoic acid, 14-methyl-, methyl ester	-3.3	-4.1	-3.9
6.	Dibutyl phthalate	-4.1	-4.5	-4.3
7.	Butylated Hydroxytoluene	-4.6	-5.1	-5.2
8.	9,12-Octadecadienoic acid, methyl ester	-4.1	-4.2	-4.5
9.	9-Octadecenoic acid (Z)-, methyl ester	-4.1	-4.3	-3.7
10.	cis-5,8,11,14,17-Eicosapentaenoic acid	-5	-4.5	-4.4
11.	Trichloroacetic acid, pentadecyl ester	-3.7	-3.6	-4.1
12.	Erucic acid	-3.7	-3.7	-3.8
13.	Linoleic acid ethyl ester	-4	-4.3	-4.4
14.	cis-Vaccenic acid	-3.8	-4.2	-4
15.	9-Eicosenoic acid, (Z)-	-3.9	-3.8	-4.6
16.	cis-11-Eicosenoic acid	-4	-4.2	-4.1
17.	4,7,10,13,16,19-Docosahexaenoic acid, methyl ester, (all-Z)-	-4.4	-3.9	-4.5
18.	Ethyl Oleate	-3.8	-4.3	-3.7
19.	2-Methyl-Z,Z-3,13-octadecadienol	-3.9	-3.9	-3.8
20.	(E)-9-Octadecenoic acid ethyl ester	-3.4	-3.9	-3.6
21.	13-Octadecenal, (Z)-	-4	-4	-4
22.	5-Octadecene, (E)-	-3.8	-3.8	-3.6
23.	3-Eicosene, (E)-	-3.9	-3.4	-3.2
24.	7,10,13-Hexadecatrienoic acid, methyl ester	-4.4	-4.4	-4.8
25.	6-Butyl-1,4-cycloheptadiene	-4.4	-4.1	-4.3
26.	i-Propyl 9-tetradecenoate	-4.1	-4	-3.9
27.	Squalene	-3.1	-5.4	-5.1
28.	Diethyl Phthalate	-4.4	-4.7	-4.3
29.	Methyl 6,9,12,15,18-heneicosapentaenoate	-4.4	-5.3	-5
30.	1-Docosene	-3.6	-4.2	-3.6
31.	Octadecanoic acid, propyl ester	-3.6	-4.2	-3.3
32.	Octadecanoic acid, ethyl ester	-3.7	-3.8	-3.7
33.	Methyl stearate	-3.5	-4.3	-3.4
34.	1-Octadecene	-3.2	-3.8	-3.8
35.	n-Propyl 11-octadecenoate	-4.1	-4.3	-4.3

Table S2: Binding Affinities (ΔG, kcal/mol) of *Cassia tora* Bioactive Compounds Against *Alternaria solani* Target Proteins (Effector Protein, Endopolygalacturonase, and Mitogen-Activated Protein).

S/No	Compounds	Binding Affinity (Kcal/mol)		
		Effector Protein	Endopolygalacturonase	Mitogen Activated Protein
1	(E)-9-Octadecenoic acid ethyl ester	-5.6	-3.9	-5.1
2	1-Docosene	-4.2	-3.5	-5.5
3	1-Octadecene	-3.7	-3.7	-5.5
4	2-Methyl-Z,Z-3,13-octadecadienol	-4	-3.7	-6
5	3-Butynylbenzene	-4.4	-4.1	-5.9
6	3-Eicosene, (E)-	-4.3	-3.3	-5.6
7	4,7,10,13,16,19-Docosahexaenoic acid, methyl ester, (all-Z)-	-5.1	-4.8	-6.8
8	5-Octadecene, (E)-	-2.8	-3.4	-5.1
9	6-Butyl-1,4-cycloheptadiene	-4.2	-4.3	-5.5
10	7,10,13-Hexadecatrienoic acid, methyl ester	-4.1	-4	-5.9
11	9-Eicosenoic acid, (Z)-	-4.2	-3.8	-5.7
12	9-Octadecenoic acid (Z)-, methyl ester	-4.6	-3.9	-4.9
13	13-Octadecenal, (Z)-	-4.1	-3.6	-5.8
14	Butylated Hydroxytoluene	-5.3	-4.7	-6
15	cis-11-Eicosenoic acid	-4.6	-4.1	-6.1
16	cis-Vaccenic acid	-4.5	-3.7	-5.9
17	Dibutyl phthalate	-4.7	-4.5	-5.6
18	Diethyl Phthalate	-5.1	-4.4	-5.6
19	cis-5,8,11,14,17-Eicosapentaenoic acid	-4.8	-4.8	-6.6
20	Ethanol, 2-(tetradecyloxy)-	-3.9	-3.8	-5.5
21	Ethyl Oleate	-3.3	-3.5	-5.6
22	Erucic acid	-4.8	-3.7	-5.6
23	Trichloroacetic acid, pentadecyl ester	-4.1	-4	-5.5
24	i-Propyl 9-tetradecenoate	-4.7	-4.1	-5.1
25	Linoleic acid ethyl ester	-3.8	-3.5	-5.9
26	Methyl 6,9,12,15,18-heneicosapentaenoate	-3.7	-3.6	-6
27	Methyl 8,11,14,17-eicosatetraenoate	-4.7	-4.4	-6.2
28	Methyl 9,12-heptadecadienoate	-4.4	-3.9	-5.3
29	Methyl stearate	-4.2	-3.8	-5.5
30	n-Propyl 11-octadecenoate	-4.5	-3.7	-5.9
31	9,12-Octadecadienoic acid, methyl ester	-3.4	-4.2	-5.7
32	Octadecanoic acid, ethyl ester	-3.9	-3.6	-5.4
33	Octadecanoic acid, propyl ester	-3.7	-3.8	-5.5

34	Pentadecanoic acid, 14-methyl-, methyl ester	-3.5	-3.5	-5.6
35	Squalene	-5.1	-4.5	-7.3
